# Integrating single-cell sQTL mapping with deep-learning splicing prediction identifies causal variants under influenza infection

**DOI:** 10.1186/s13059-026-04205-9

**Published:** 2026-07-18

**Authors:** Liuyang Wang, Guinevere G. Connelly, Trisha Dalapati, Angela G. Jones, Benjamin H. Schott, Joseph D. Trimarco, Nicholas S. Heaton, Dennis C. Ko

**Affiliations:** 1 Department of Molecular Genetics and Microbiology, School of Medicine, Duke University, 0048B CARL Building Box 3053, 213 Research Drive, Durham, NC 27710 USA; 2 Duke University Program in Cell and Molecular Biology, Duke University, Durham, NC USA; 3 Duke University Program in Genetics and Genomics, Duke University, Durham, NC USA; 4 Duke Center for Virology, Duke University, Durham, NC USA; 5 Department of Medicine, School of Medicine, Duke University, Durham, NC USA

**Keywords:** Hi-HOST, Cellular GWAS, 1000 Genomes, Isoform QTL, Pangolin, COVID-19, HSV-1, OAS1, U2AF1L4, IFNAR2

## Abstract

**Background:**

Realizing the full potential of human genetics requires identifying causal variants and genes underlying association signals. Molecular quantitative trait locus (molQTL) analyses, such as expression QTL (eQTL) and splicing QTL (sQTL), link genetic variants to intermediate molecular phenotypes, but pinpointing causal variants and their regulatory effects remains challenging. Here, we integrate sQTL analysis with deep learning-based splicing effect annotation to identify causal genetic variants and elucidate their functional effects on human phenotypes.

**Results:**

Applying a single-cell GWAS method, scHi-HOST, across 96 lymphoblastoid cell lines with and without influenza A virus (IAV) infection, we identify approximately 43,000 sQTL SNP-junctions pairs associated with 217 genes during infection. Integrating sQTL mapping with AI based splice prediction and statistical fine-mapping, we prioritize 57 likely causal variants that affect cis-acting molecular splicing components, including 5’ donor and 3’ acceptor sites. We experimentally validate one such variant, rs2297616, in *PARP2*, encoding poly (ADP-ribose) polymerase 2. This variant alters the 5’ splice donor site in the second intron of *PARP2*, generating two protein isoforms differing by 13 amino acids. The derived A allele is associated with the longer protein isoform and increased IAV levels in lymphoblastoid cell lines. CRISPR mediated editing validates the causal effect of this variant on both protein length and IAV infection. Furthermore, these 57 putative causal sQTLs are linked to over 100 GWAS traits, including many variants associated with autoimmune diseases.

**Conclusions:**

Our work provides a catalog of causal sQTL with direct splicing impacts, providing causal mechanistic insights from genotype to disease susceptibility.

**Supplementary Information:**

The online version contains supplementary material available at https://doi.org/10.1186/s13059-026-04205-9.

## Background

Deciphering the causal genetic basis of disease susceptibility is central to human genetics and precision medicine [1]. Molecular quantitative trait loci (molQTLs), e.g. expression QTLs (eQTLs) and splicing QTLs (sQTLs), provide potential mechanistic insight by linking genetic variants to intermediate molecular phenotypes [2–4]. Further, joint analysis of GWAS and molQTL has shown promise in narrowing down candidate causal SNPs and their underlying causal genes [5, 6].

Two broad categories of joint analyses have been widely used to prioritize disease-associated genes. The first, exemplified by PrediXcan [7] and transcriptome-wide association studies [8], estimate association of imputed gene expression and disease outcomes. A complementary approach relies on colocalization to infer shared causal genetic signals between GWAS and molQTL in a specific genomic region (e.g. coloc [9], RTC [10], Sherlock [11], and eCAVIAR [12]). Despite their success in pinpointing causal factors, the first type of method focuses on identifying causal genes (and not causal variants) that impact bulk expression levels, while the latter focuses on identifying causal variants without revealing the underlying mechanisms. Despite these advances, pinpointing causal disease variants, particularly when multiple candidate SNPs are in high linkage disequilibrium (LD), and how they impact causal genes remain challenging.

In this study, we combined sQTL analysis and deep learning-based AI variant annotation to discover causal variants that directly affect splicing. Ninety percent of human genes encode for alternatively spliced transcripts [13], and mutations at splice sites can directly alter expression of transcripts and protein isoforms. It is estimated that up to 60% of rare disease-causing variants exert their pathogenic effects by disrupting normal splicing patterns [14], while the contribution of common variants that affect splicing to complex traits and diseases is comparable to, or even larger than, that of eQTLs [15–17].

Past work has identified sQTLs at baseline in lymphoblastoid cell lines (LCLs) [18], other cell types [3, 19], and complex tissues [20, 21]. Unlike eQTLs, which typically capture aggregated gene-level expression changes, sQTLs can reveal effects of genetic variation on specific molecular events that regulate individual splice junctions that control protein isoforms. However, most sQTL studies do not integrate sQTL identification with systematic variant annotation that reveals causal variants and underlying splice mechanisms.

A major factor that has hampered our understanding of how SNPs affect splicing is incomplete functional annotation, such as undocumented splice events and inaccurate prediction of splice sites. In understanding the effects of rare variants, this has previously left the majority of variants overlapping predicted splice sites categorized as variants of unknown significance (VUS) [22]. Recent application of deep learning algorithms and genome sequences to predict splicing variants, such as SpliceAI [23] and MMSplice [24], has revolutionized the interpretation of rare variants. These tools utilize convolutional neural networks (CNNs) and the genomic context of the surrounding DNA sequence of each focus variant to predict the potential of altering transcript isoform. Newer tools, including Pangolin [25] and AbSplice [26], incorporate both DNA- and transcriptome- based models to predict variant splicing score across different tissues. In 2023, AI-based splicing prediction was recommended as a key criterion for variant pathogenic evidence by the ClinGen SVI Splicing Subgroup [27] for diagnosis of rare and severe genetic disease [28]. Thus, the use of AI in interpretation of rare variants has proven highly successful.

We reasoned that sQTL analysis and AI variant annotation are complementary methods that could pinpoint common causal disease variants. While sQTLs capture the regulatory effects of genetic variants across large LD-linked regions, deep-learning-based AI models predict splice-altering potential at single-nucleotide variant resolution. By comparing predicted splicing effects within sQTL association signals, the variants with the strongest predicted impact on splice site usage can be prioritized as putative causal variants.

To test this concept, we adapted our previously developed single-cell GWAS platform (scHi-HOST), which combines single cell RNA-seq (scRNA-seq) and GWAS to rapidly identify eQTLs and SNPs that confer resistance and susceptibility to any stimulus [29]. In this study, we used influenza A virus (IAV), a seasonal epidemic virus and significant burden to global public heath that infects approximately 30 million people every year, with 290,000–650,000 deaths [30]. A previous study demonstrated that IAV infection outcome is heritable [31], and a recent large-scale GWAS (> 270,000 individuals) reported two loci associated with influenza infection [32]. Further, previous work has demonstrated the importance of splicing changes during IAV infection [33–36]. These findings highlighted the important but underexplored role of host genetics and splicing in influenza pathogenesis.

In extending scHi-HOST to identify causal sQTLs, we identified > 43,000 sQTL SNP-junction pairs during IAV infection and used AI-based splice annotation and statistical fine mapping to connect 26% of sJunctions (junctions associated with an sQTL) to a causal variant with a predicted mechanism of action. A predicted causal variant within the poly (ADP-ribose) polymerase 2 (*PARP2*) gene creates a new splice donor site, resulting in a longer protein isoform with 13 additional amino acids. Amplification of cDNA, sequencing, and western blots of CRISPR-edited cells revealed different isoform expression of PARP2 as predicted. Additionally, scHi-HOST data showed that rs2297616 is associated with IAV burden, suggesting that PARP2 isoform usage regulates IAV infection—a finding confirmed with CRISPR-edited cells. This same variant is associated with herpes simplex virus 1 (HSV-1) infection in UK Biobank data [37], pointing to a more general role in viral infection. More broadly, the causal sQTLs identified by our study are highly enriched in human disease GWAS, highlighting the value of integrating sQTLs and AI for identification of causal variants in human disease.

## Results

### scHi-HOST reveals global changes in splicing induced by IAV and reproducible sQTLs in uninfected and IAV-infected states

**Fig. 1:**
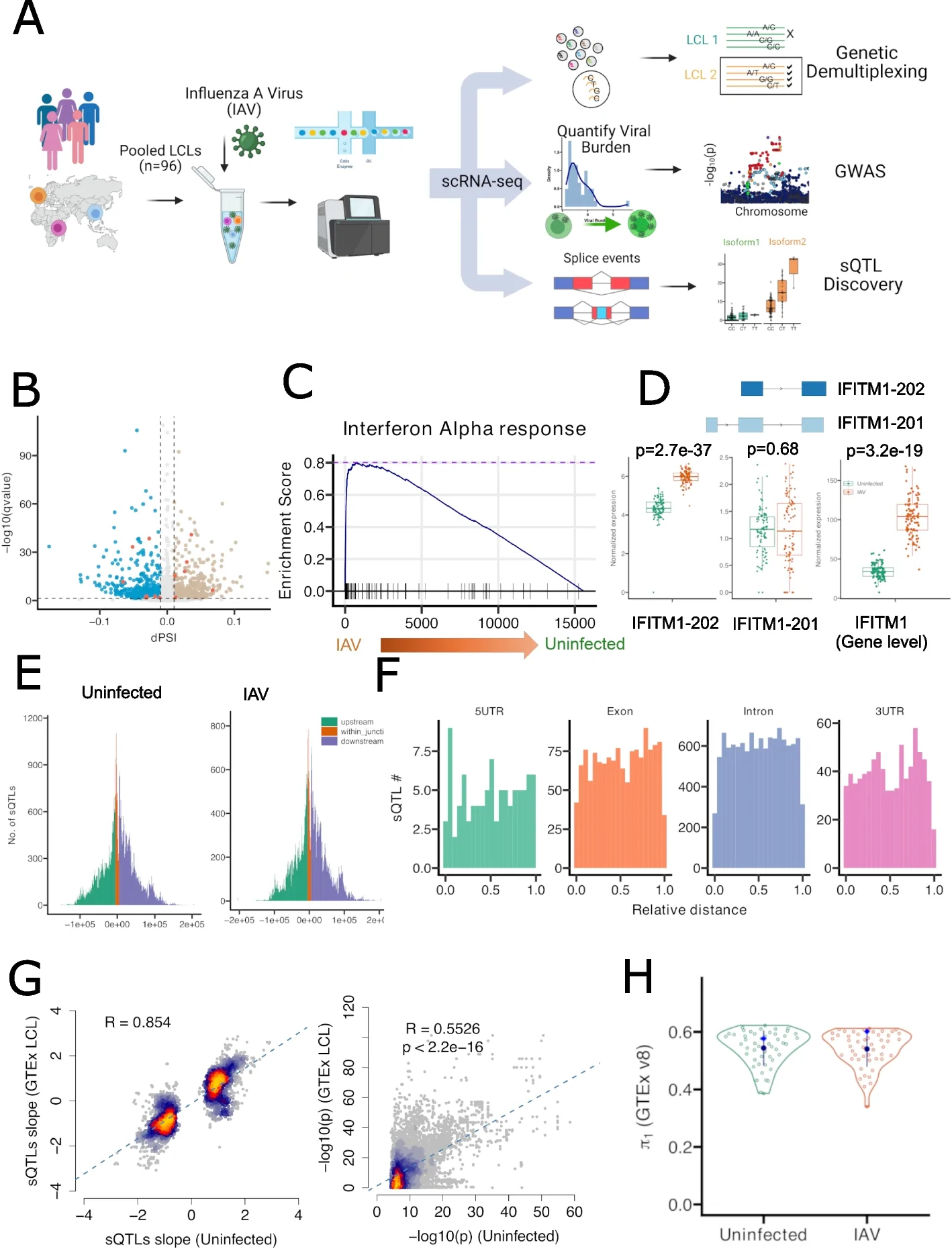
Characterization of global transcript changes after IAV infection and discovery of sQTLs based on scHi-HOST splicing platform. **A** Flowchart of scHi-HOST sQTL pipeline. **B** Volcano plot of differentially spliced junctions between uninfected and IAV-infected. Each dot represents a splice junction. The bisque-colored dots indicate significantly higher usage of junctions after IAV (dPSI > 0.01), and blue dots indicate significantly lower usage of junctions after IAV (dPSI < −0.01), gray dots depict junctions that are not significantly different. Orange dots represent splice junctions from genes in the interferon alpha response pathway. **C** GSEA plot shows significant enrichment of interferon alpha response pathway genes after IAV infection. **D** The major transcripts of *IFITM1* from the interferon alpha response pathway and boxplots of *IFITM1* expression at the transcript level (left) and gene level (right). **E** The distribution of sQTLs relative to the splicing junction. Density of sQTL-SNPs upstream of the junction start is in green with distance relative to the upstream junction coordinate, while density of sQTL-SNPs downstream of the junction is in blue with distance relative to the downstream junction coordinate, and density of sQTL-SNPs between the junction coordinates (located within the spliced intron) are shown in orange. The junction length was normalized to 10,000 bp. The majority of identified sQTLs are located near the junction starting or ending site and decrease with distance. **F** Distribution of sQTLs across four major gene features. 5’ UTRs, exons, introns, and 3’ UTRs for MANE (Matched Annotation from NCBI and EMBL-EBI) transcripts were scaled from 0 to 1 (to indicate the beginning and end of each feature) and divided into 20 bins. Each sQTL was placed in a bin based on its relative location. **G** Replication of scHi-HOST sQTLs in GTEx. The left panel shows slope comparison of significant sQTL-SNPs from the uninfected scHi-HOST condition (y-axis) and GTEx EBV-infected LCL (x-axis), and *p*-value comparison (right panel). **H** Boxplot of ${\pi}_{1}$ statistics indicates scHi-HOST are highly reproducible in GTEx 49 tissues for both Uninfected and IAV

To characterize the global transcriptional response to IAV infection, we conducted alternative splicing analysis and sQTL discovery in an IAV-infected LCL model based on our single-cell GWAS platform, scHi-HOST [29]. The scHi-HOST workflow consisted of five key components: 1) infection of 96 pooled LCLs from six 1000 Genomes populations with the CA09 IAV strain, followed by scRNA-sequencing using 10X Genomics Chromium 3’ kit, 2) assignment of each singlet to its corresponding LCL by genetic demultiplexing [38], 3) mapping and quantification of reads for generation of pseudobulk phenotypes, 4) association analysis on viral burden phenotypes, and 5) sQTL discovery with Leafcutter [16] (Fig. 1A). For this study, we recovered 31,155 singlets for the uninfected condition, 13,794 singlets for the IAV condition.

To quantify splicing changes during IAV infection, we counted intron clusters using Leafcutter and identified 33,315 junctions in the uninfected condition and 28,366 junctions under IAV infection. Further differential splicing analysis identified 2,151 junctions from 1,563 genes that were significantly altered between IAV and uninfected conditions (q-value < 0.05 and |ΔPSI|> 0.01) (Fig. 1B; Additional file 1: Table S1). Of significant differentially spliced junctions, ~ 62% resulted from exon-skipping events (Additional file 2: Fig. S1). Gene set enrichment (using Enrichr [39–41]) for genes that harbor significant differentially spliced junctions revealed strong enrichment of interferon-related pathways after IAV infection, including interferon alpha response (Fig. 1C; Additional file 2: Fig. S2; Additional file 3: Table S2). Elevated interferon alpha and gamma responses were visualized at the single-cell level using UMAP plots (Additional file 2: Fig. S3). Consistent with previous findings [42], we found significantly elevated aggregated *IFITM1* expression after IAV infection (*p* = 3.2e-19) (Fig. 1D; Additional file 2: Fig. S4). At the transcript isoform level, the canonical isoform *IFITM1-202*, was dramatically upregulated after infection relative to uninfected controls (*p* = 2.7e-37). However, the alternative primary isoform, *IFITM1-201*, showed no significant change (*p* = 0.68) (Fig. 1D). Thus, IAV infection induces higher expression of *IFITM1* by selectively upregulating the *IFITM1-202* isoform rather than the *IFITM1-201* isoform. These findings underscore how transcript analysis enables the capture of isoform-specific changes during pathogen infection.

We used Leafcutter to identify sQTLs that regulate splicing in uninfected and IAV-infected conditions. From uninfected LCLs, we identified 53,292 sQTL SNP-junction pairs from 597 junctions and 317 genes, and from IAV-infected LCLs, we found 43,015 sQTL SNP-junctions affecting 411 junctions from 217 genes (FDR < 0.05; Additional file 2: Fig. S5; Additional file 4: Table S3). Unsurprisingly, increased read depth enhanced detection: genes with high expression showed increased probability of detecting sQTLs, peaking at around 10,000 reads (Additional file 2: Fig. S6). Therefore, we incorporated library size as a covariate in our sQTL pipeline. Note that mean read depth per gene was higher for uninfected (366) compared to IAV-infected conditions (283), and a greater number of singlets were analyzed in uninfected as noted above. Both factors likely resulted in greater power to detect sQTLs in uninfected LCLs in our dataset.

We examined the genomic distribution of sQTL-SNPs relative to their target splice junctions (sJunctions). As expected, density distribution showed that most sQTL-SNPs from both conditions were located close to the junction site and decreased with relative distance (Fig. 1E). 51.2% of uninfected and 51.8% of IAV sQTL-SNPs were within 10 kb of alternative splice event, consistent with previous studies that reported that two-thirds of sQTLs fall within a 10 kb window [34]. We next mapped all sQTL-SNPs to gene features and visualized their positions after normalizing across the relative length of each gene feature. sQTL-SNPs were detected most in introns, followed by intergenic regions, exons, 3’ UTRs, and 5’ UTRs (Fig. 1F; Additional file 2: Fig. S7). Within each feature, sQTL-SNPs were found at similar density throughout the feature type.

To assess the reproducibility of our identified sQTLs, we compared them to sQTLs identified by GTEx [3]. We found both effect size (β) and *p*-values of matched sQTL SNP- junction pairs were consistent between our uninfected condition and GTEx EBV-transformed LCLs (R^2^ = 0.854, *p* < 2.16e-16 for β comparison; R^2^ = 0.55, *p* < 2.16e-16 for *p*-values) (Fig. 1G; Additional file 2: Fig. S8). We further evaluated the replication of uninfected and IAV sQTL-SNPs using the ${\pi}_{1}$ statistic against all 49 GTEx tissues. We found intermediate-high ${\pi}_{1}$ values for both conditions (Fig. 1H) (median value 0.56 for Uninfected, 0.56 for IAV), indicating many of our identified sQTLs were also detected in GTEx.

### sQTLs identified during IAV infection are enriched for genes involved in interferon signaling and can be constitutive, responsive, or induced

**Fig. 2:**
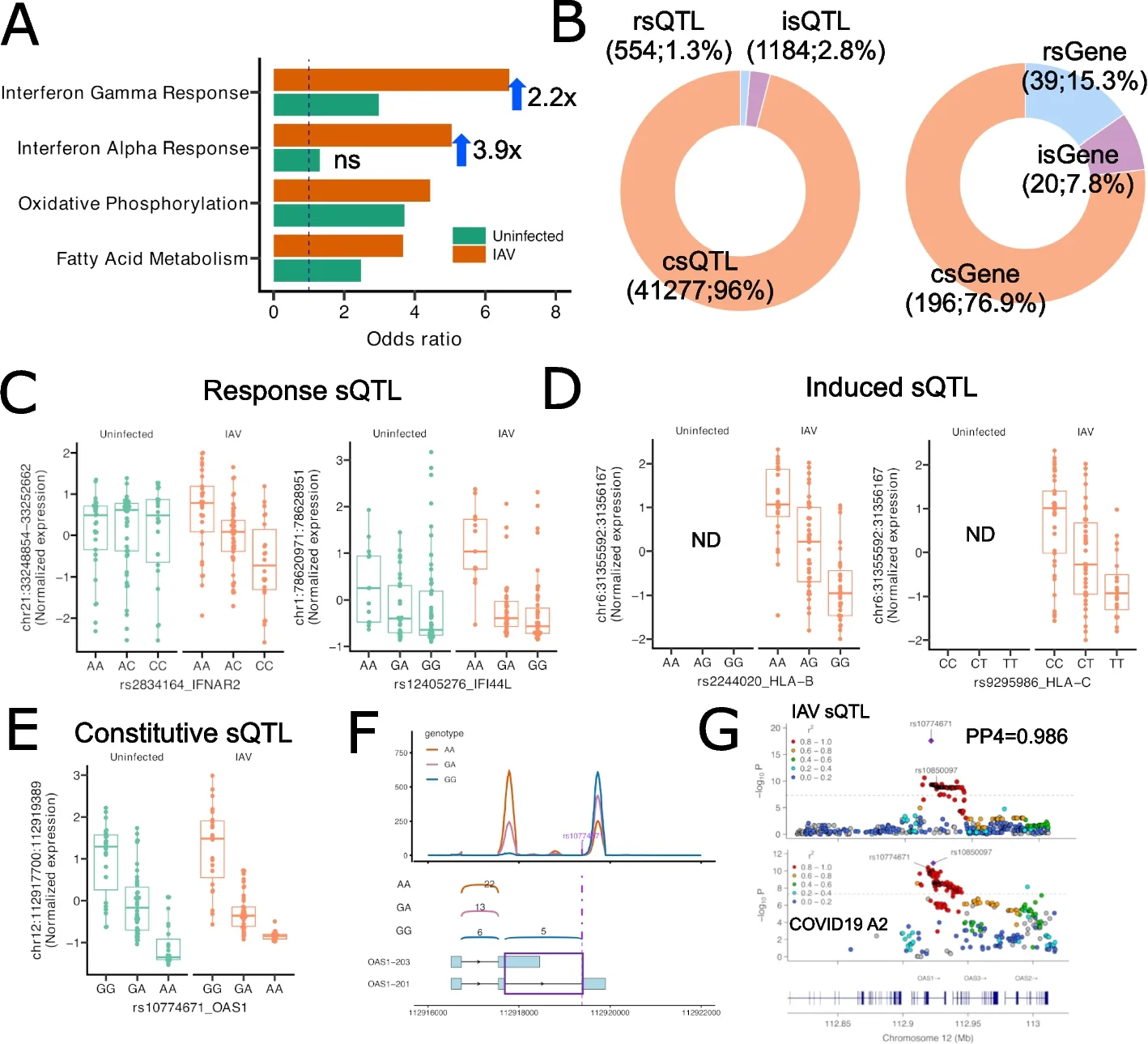
Classification of scHi-HOST IAV sQTLs. **A** Enrichment analysis of MSigDB Hallmark gene sets based on detected sGenes. Four gene sets at FDR < 0.1 were identified during IAV infection, including interferon gamma and alpha response pathways. **B** Donut plots illustrating the distribution of IAV sQTLs (left) and sGenes (right). The sQTLs are categorized into three broad groups defined in the text: IAV-responsive sQTLs (rsQTL), induced sQTL (isQTL), and constitutive sQTLs (csQTLs). **C** Two examples of rsQTLs. Left, SNP genotypes show minimal correlation with normalized junction expressions under uninfected conditions (β = 0.007, *p* = 0.94 for *IFNAR2*; β = −0.36, *p* = 0.018 *IFI44L*) but exhibit significant correlation under IAV-infected conditions (β = −0.69, *p* = 6.3e-7 for *IFNAR2*; β = −0.63, *p* = 2.6e-6 *IFI44L*). **D** Two examples of isQTLs from the HLA region. The junctions were detected with IAV infection but not detected (ND) in uninfected. For *HLA-B*, β = −0.91, *p* = 3.8e-11 IAV-infected. For *HLA-C*, β = −0.82, *p* = 2.3e-8 IAV-infected. **E** One example of constitutive sQTL from *OAS1*, with β = −0.96, *p* = 8.9e-14 uninfected, and β = −1.05, *p* = 2.7e-18 for IAV-infected. **F** Sashimi plot showing read coverage (top) and junction usage (middle) at *OAS1* stratified by rs10774671. The junction highlighted with purple rectangle matches to E. **G** LocusZoom plots of the *OAS1* locus for IAV sQTL (top) and GWAS in severe/critical COVID19 (A2) (bottom). Coloc revealed a PP4 of 0.986, suggesting strong evidence of a shared causal variant

We performed gene enrichment analysis on sGenes to characterize the biological process underlying identified sQTLs based on the MSigDB Hallmark gene set. Four gene sets showed significant enrichment at an FDR level of 0.1, with three of these also showing significant enrichment in uninfected LCLs (Fig. 2A; Additional file 5: Table S4). Three gene sets, interferon gamma response, oxidative phosphorylation, and fatty acid metabolism, were significant under both conditions, although interferon gamma response showed a 2.2 × greater enrichment with IAV infection. Interferon alpha response showed even greater enrichment during IAV infection and was not significantly enriched in the uninfected state. The sGenes found within this gene set include MX1, HLA-C, and IFI44, which play important roles in host response specifically to viral infections. These findings highlight genetic control of splicing in key innate immunity genes that becomes apparent only in the context of infection.

sQTLs identified during IAV infection could be constitutive, responsive, or induced. To differentiate sQTLs that are constitutive compared to those whose effect size changes significantly with infection, we used a unified genotype-by-condition interaction model. We conducted a linear mixed-effects model (LMM) analysis in which quantile-normalized junction usage was modeled as a function of genotype, treatment, covariates, and donor as a random effect. A likelihood ratio test (LRT) was used to test for significant genotype x treatment interaction. Among the 43,015 sQTL SNP-junction pairs detected during IAV infection, 1.3% (*n* = 554) showed a significant difference with q-value < 0.1 from LRT test and were therefore defined as IAV response sQTL-SNPs (rsQTL-SNPs). For 2.8% (*n* = 1184) of sQTL-sJunction pairs, the specific sJunction was not detected in the uninfected condition. Such induced sQTL-SNPs (isQTL-SNPs) are associated with splicing events that appear to only occur during infection. Of these, 607 induced rsQTL-SNPs have no matched junctions in uninfected, while the other 577 have extremely low expression of matched junctions and were therefore filtered out by Leafcutter’s default filtering parameter. The remaining 96% (*n* = 41,277) were defined as constitutive sQTL-SNPs (csQTL-SNPs) (Fig. 2B). The 554 rsQTL-SNPs affect splicing of 39 rsGenes (15.3%), and 1184 induced sQTL-SNPs impact 20 genes (7.8%), while the 41,277 csQTL-SNPs affect 196 csGenes (76.9%) (Fig. 2B; Additional file 6: Table S5), demonstrating a substantial number of sQTLs exhibit IAV-responsive regulation. Examples of rsQTL and isQTL, which are also within the interferon response genesets, are plotted in Fig. 2C and D. As a less stringent alternative to the LRT interaction approach, we also performed a post-hoc z-test that compares the slopes of matched sQTL SNP-junctions, followed by FDR correction (Additional file 2: Fig. S9). This more exploratory approach revealed 1,033 rsQTL-SNPs with the caveat that there are likely more false positives in this set (Additional file 6: Table S5).

The emergence of sQTL in interferon genesets during IAV infection suggested that such variants might also be associated with viral infection in humans. We determined whether any of the csQTL, rsQTL, and isQTL overlapped with human GWAS of influenza or severe COVID-19. A large GWAS of influenza infection based on self-reported positive flu test over a 2-year period revealed two genome-wide significant SNPs [32], but neither overlapped with an sQTL signal from our dataset. In contrast, two sQTL were also associated with severe COVID-19 (COVID19hg A2 phenotype (rel7 [43]). The first is a well-characterized csQTL for *OAS1* (rs10774671; Fig. 2E, F) that was first identified prior to the GWAS era [44] and has now been mechanistically linked to severe COVID-19, as well as West Nile Virus [44–51]. Our csQTL signal for *OAS1* colocalizes with the signal for severe COVID-19 (Fig. 2G; PP4 = 0.986). It has been previously reported that the A allele of rs10774671 results in expression of only the p42 isoform (OAS1-203) of OAS1, while the G allele results in expression of both the p46 (OAS1-201) and p42 isoforms [44]. The p46 isoform is prenylated which has been shown to be necessary for OAS1 dsRNA sensing of SARS-CoV-2 [49]. The second is an rsQTL-SNP for the interferon receptor IFNAR2 (rs2834164; see Fig. 2C) that is in LD with a variant associated with severe COVID-19 (rs2236757; r^2^ = 0.50 in 1000 Genomes populations in LDlink [52]) [48]. As this moderate LD would suggest, the two signals do not appear to colocalize (Additional file 2: Fig. S10A; PP4 = 0.016). The COVID-19 association signal here appears complex, though another SNP in LD (rs13050728; r^2^ = 0.55) has been reported as an eQTL for *IFNAR2* within monocytes [53], and a recent publication indicates that a different sQTL (rs2284550; r^2^ = 0.99) from blood does demonstrate colocalization with hospitalized COVID-19 [54] (COVID19hg B1 phenotype). Indeed, our sQTL-SNP demonstrates colocalization with the B1 phenotype as well (Additional file 2: Fig. S10B; PP4 = 0.995).

### Integration of AI with sQTLs reveals putative causal SNPs

We identified a total of 61,216 sQTL SNP-junction pairs, which were associated with only 721 unique splice junctions from 378 sGenes. While typically many sQTL-SNPs in high LD are associated with each splicing event, we hypothesized that each independent signal would be driven by a single causal sQTL.

To identify causal variants associated with each splice event, we applied two state-of-the-art AI-based splicing prediction tools, SpliceAI [23] and Pangolin [25]. Both AI models employ convolutional neural networks to estimate splicing changes by comparing predicted splice potential between reference and alternative alleles based on local genomic sequence context. SpliceAI predicts the probability that a nucleotide substitution alters canonical donor or acceptor sites, providing four scores: acceptor gain/loss and donor gain/loss. Pangolin uses a similar deep-learning architecture but predicts splice-site usage with tissue specific context, outputting two probabilities (gain or loss) without distinguishing donor from acceptor sites. Both tools have demonstrated superior accuracy compared to other methods [55], with SpliceAI providing clear donor/acceptor gain/loss interpretability and Pangolin offering tissue-aware prediction and multi-species training data. Therefore, we integrated both methods to obtain a robust set of casual splice variants with a clear mechanistic prediction.

**Fig. 3:**
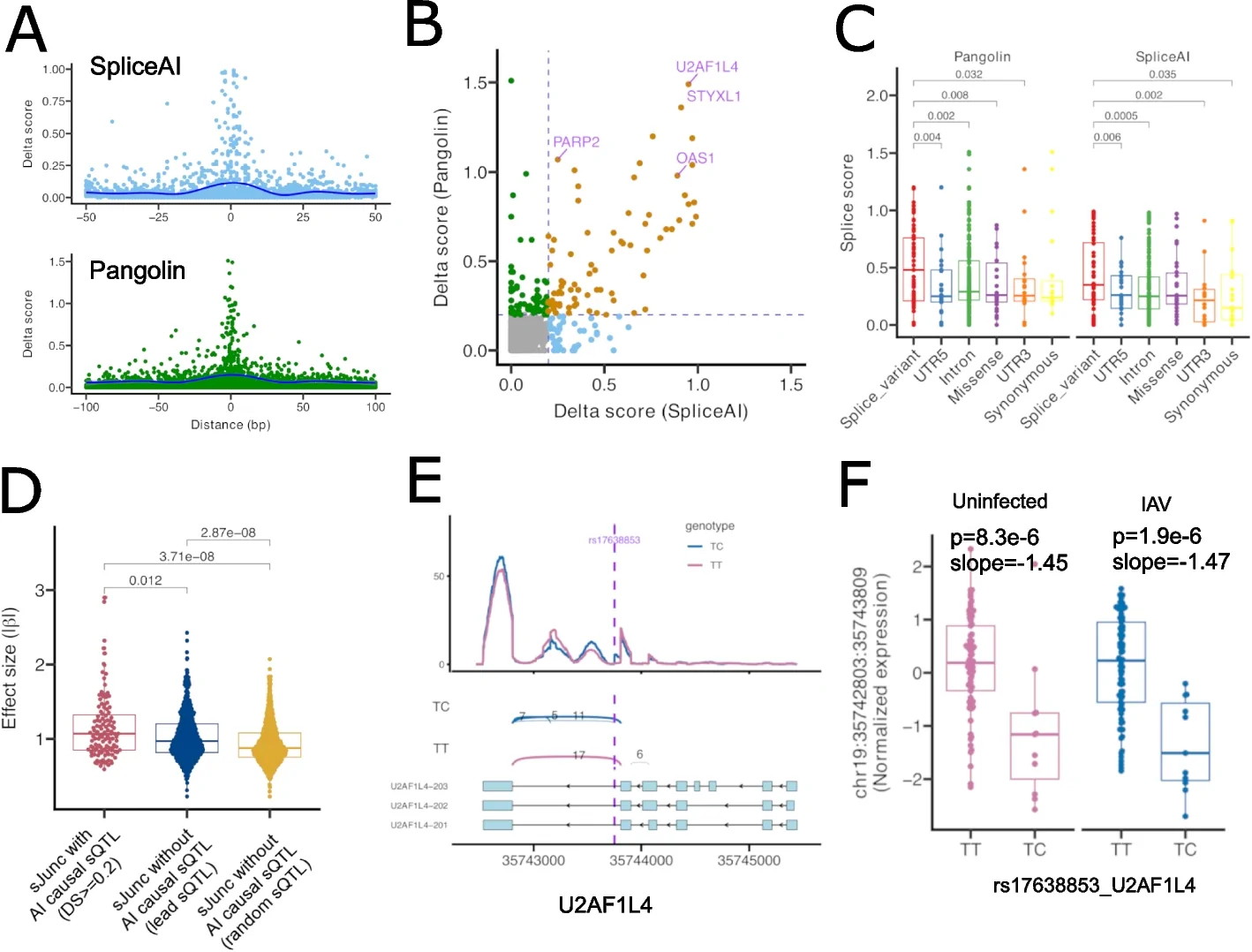
Integration of sQTL identification with AI to identify putative causal variants. **A** The distribution of SpliceAI (top) and Pangolin delta score (bottom) for scHi-HOST sQTLs plotted based on their location relative to the splice junction. **B** Scatter plot of SpliceAI delta_score against Pangolin delta_score. The vertical and horizontal line indicate a cutoff of 0.2. The dot color indicates those that are shared (orange), SpliceAI-specific (blue), or Pangolin-specific sQTLs (green). **C** Boxplot of sQTL splice scores across different groups. The *p*-values are from the Wilcoxon rank sum test. **D** Boxplot of absolute β values of sQTL-SNPs from sJunctions with vs without AI-predicted causal variants. **E** Sashimi plot of *U2AF1L4* from pseudo-bulk data stratified by rs17638853 genotype. Exon reads are represented by the peaks in the top, and junction reads are represented by arcs in the middle. **F** Genotype boxplot of rs17638853 against normalized *U2AF1L4* junction counts. Boxplots show median and interquartile range, with each dot representing normalized junction read of a single LCL

As expected, higher delta scores (DS) from both methods were assigned to sQTL-SNPs closer to the splice junction, especially within the splice site (distance = 0) or ± 10 bp for both SpliceAI and Pangolin (Fig. 3A). A direct comparison of the two methods showed high concordance along with some algorithm-specific variants (Fig. 3B). Categorization by variant annotation from Ensembl VEP (Variant Effect Predictor) v106 demonstrated that sQTL-SNPs overlapping with known splice sites had the highest predicted splice scores from both tools, significantly higher than other annotated groups, such as 3’ UTR and 5’ UTR (Fig. 3C).

By adopting a DS cutoff of 0.2 from previous studies [23, 25] for both SpliceAI and Pangolin, we identified 76 AI-predicted causal sQTL-SNPs from 28,674 unique sQTL-SNPs. These predicted causal variants were associated with 67 unique sGenes and 119 sJunctions, indicating that these AI tools agree on the likely causal variant for 18% of sGenes and 17% of sJunctions (Additional file 7: Table S6). If we took a less stringent approach by requiring that only one of the tools have a DS ≥ 0.2, then 38% of sGenes and 37% of sJunctions had an AI-predicted causal variant.

Thus, AI was able to assign a predicted causal variant to nearly 40% of sQTL signals. However, this was still substantially less than the 70–90% sensitivity reported in using these same tools to classify rare, splice-disrupting disease variants [27, 55]. We hypothesized that unlike the rare splice-disrupting variants associated with severe disease, common sQTLs likely have less severe effects on splicing and thus would be more challenging for deep learning models to identify. We grouped all sJunctions as having a likely causal variant assigned by AI (SpliceAI and Pangolin scores ≥ 0.2) or not. Comparing the effect sizes (absolute value of β) of AI-predicted causal variants vs. randomly selected sQTL-SNPs for each sJunction demonstrated that cases where a causal variant was predicted by AI displayed a larger effect size (Fig. 3D). As the AI-prediction is made independently of effect size, the comparison to randomly select sQTLs was considered appropriate, but causal variant effect sizes were still significantly greater even when compared to lead variants for sJunctions with no strong AI-predicted causal variant. This indicated that these deep learning methods are more likely to identify causal variants for sQTL association loci that have more severe splicing effects (larger effect sizes).

Among the top-ranked variants, rs17638853 had a DS of 0.95 by SpliceAI and of 1.49 by Pangolin. This variant is located within the last intron of *U2AF1L4* (U2 small nuclear RNA auxiliary factor 1-like 4, a paralog of *U2AF1*), which encodes a splicing factor that is part of the heterodimeric complex that recruits the U2 snRNP to the polypyrimidine tract and AG dinucleotide at the 3′ splice site. The alternative allele C creates a novel 5' donor site based on Sashimi plot and SpliceAI prediction (Fig. 3E), creating a previously unknown isoform that is 19 amino acids longer. This was further supported by the AlphaGenome [56] prediction that the alternative allele C is exclusively associated with a shorter junction in three different tissues (Lung, EBV-transformed LCL, and Whole blood (Additional file 2: Fig. S11). rs17638853 is a csQTL, as the heterozygous genotype TC was significantly correlated with lower normalized junction ratio in both uninfected and IAV states (Fig. 3F).

Similarly, a causal variant for an sQTL signal for *OAS1* was also identified by both methods (rs10774671; DS of 0.89 by SpliceAI and of 0.98 by Pangolin). This is the known causal variant that determines *OAS1* exon usage and is associated with severe COVID-19 (see Fig. 2E, F). Collectively, these findings demonstrate the utility of AI-driven splicing predictions in refining sQTL signals to pinpoint causal variants, particularly those of larger effect size.

### Most AI-predicted causal variants fall within 95% credible sets from statistical fine mapping

**Fig. 4:**
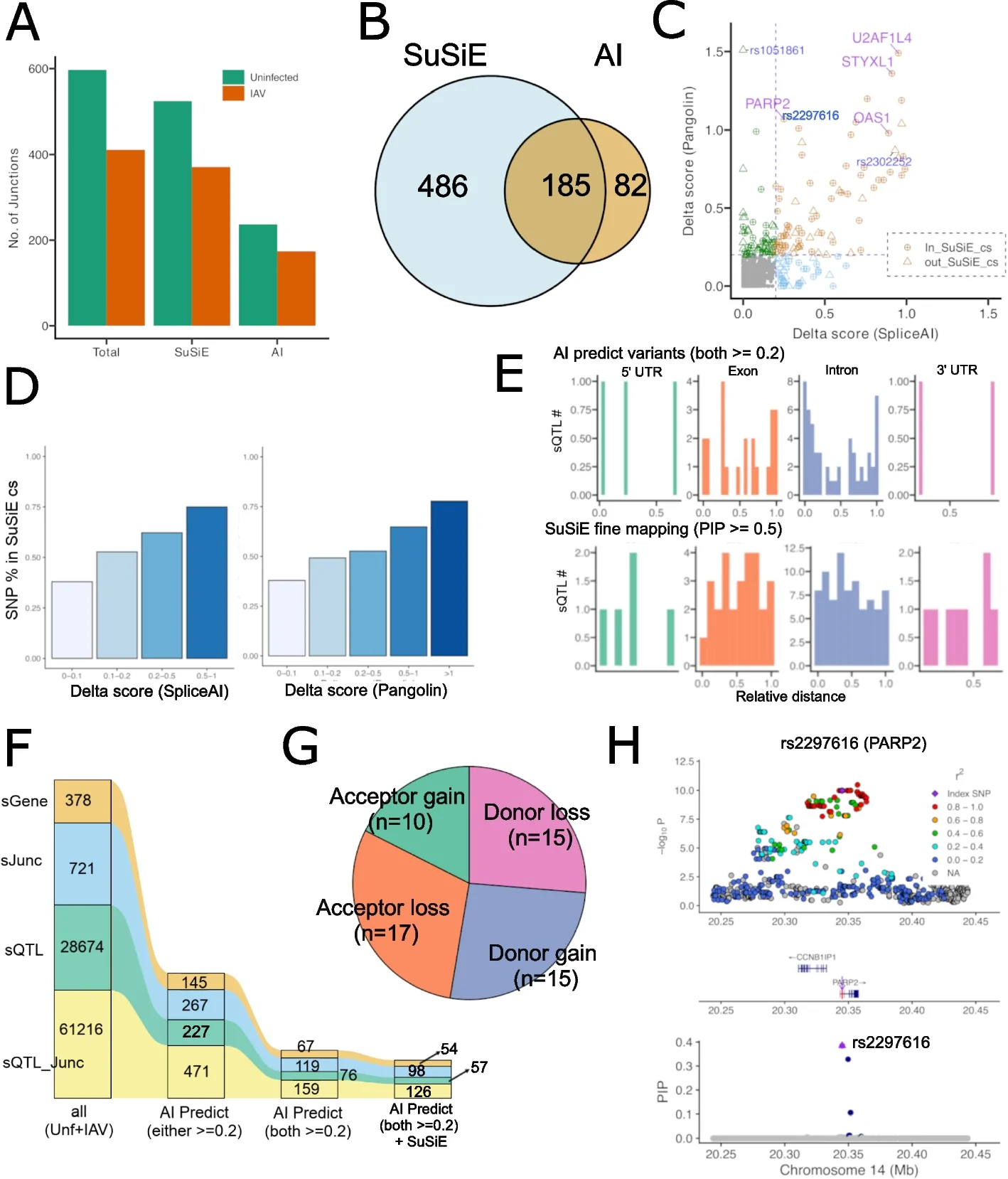
Comparisons of AI-based splice prediction and SuSiE fine-mapping reveals a common set of high-confidence predicted causal variants. **A** Number of splice junctions in uninfected and IAV conditions across all sQTLs, within SuSiE fine-mapping loci with 95% credible sets (CS), and loci harboring AI-prioritized variants (either score ≥ 0.2). **B** Venn diagram showing a significant overlap between junctions from SuSiE fine-mapping 95% CS and those harboring AI-prioritized variants (either score ≥ 0.2) (OR = 3.3, *p* = 6.5e-18 from Fisher’s exact test). **C** Scatter plot of SpliceAI versus Pangolin splice scores, with point shape indicating if the variant falls in SuSiE 95% CS (circled plus) or not (triangle). Representative variants that fall outside a 95% CS and described further are labeled in blue. **D** Fraction of AI-predicted causal variants within SuSiE CS across different score bins of SpliceAI (left) and Pangolin (right). **E** Distribution of AI-predicted causal sQTLs (top) and SuSiE fine mapping sQTLs (PIP ≥ 0.5) (bottom) across 4 major gene features. 5’UTRs, exons, introns, and 3’ UTRs for MANE (Matched Annotation from NCBI and EMBL-EBI) transcripts were scaled from 0 to 1 (to indicate the beginning and end of each feature) and divided into 20 bins. Each sQTL-SNP was placed in a bin based on its relative location. **F** River plot summarizes the number of sQTL, sGenes, sJunctions. Based on SuSiE and a cutoff of 0.2 for both SpliceAI and Pangolin, we identified 57 causal sQTLs. **G** The mechanism of the 57 causal variants assigned by SpliceAI. **H** LocusZoom plot of *PARP2* sQTL, with top panel showing sQTL association, middle showing gene tracks with variants (purple arrow) and sJunction (red), and bottom panel showing SuSiE PIP scores

We then compared the AI-predicted causal sQTL-SNPs to statistical fine-mapping results using SuSiE [57]. Statistical fine mapping with SuSiE uses a Bayesian sum-of-single-effects model to calculate a probability that a variant explains the overall association signal. For a particular signal, the 95% credible set (CS) contains the variants that together have a 95% probability of containing at least one causal variant. Most sJunctions from scHi-HOST have a 95% CS, indicating that most signals appear to have a statistical causal variant (based only on statistical association and LD), but substantially fewer have an AI-predicted biological causal variant (Fig. 4A). Comparing the two demonstrated that for 267 sJunctions with an AI-predicted causal variant (AI score ≥ 0.2 for either SpliceAI or Pangolin), the matched sQTL-SNP was also within the 95% CS for 185 (69%) (Fig. 4B). This means predicted causal variants supported by AI and SuSiE can explain 26% of sJunctions. The higher the AI score, the more likely the variant fell within a 95% CS (Fig. 4C, D). Further, AI-predicted causal variants follow more of a U-shaped distribution in exons and introns, consistent with causal variants being located near known splice junctions (Fig. 4E; Additional file 2: Fig. S12; compare to Fig. 1F). Notably, the distribution still displayed many putative causal variants far from known junctions, which may reveal previously unknown junctions or splicing enhancers. sQTL-SNPs with high posterior inclusion probability (PIP ≥ 0.5) within each 95% CS did not cluster at the ends of exons/introns as observed for AI-putative causal variants (and similar to the distribution of lead variants) (Fig. 4E; Additional file 2: Fig. S12). This might be expected given the small size of the scHi-HOST dataset (*n* = 96). While SuSiE is robust, its PIPs are highly dependent on the precision of the underlying association statistics and the smaller size of our dataset increases the risk that the model identifies spurious signals driven by noise and outliers. Therefore, we combined AI prediction (both ≥ 0.2) and SuSiE fine-mapping and identified 57 high-confidence causal sQTLs, associated with 98 splice junctions from 54 sGenes (Fig. 4F; Additional file 8: Table S7). We feel these dual threshold criteria are appropriate given the study size, but we also explored the use AI scores as priors within the SuSiE framework (Additional file 2: Fig. S13), as others have done with other functional annotation [58, 59]. Further mechanistic classification of the 57 predicted causal variants revealed a slightly higher number of acceptor loss (*n* = 17, 29.8%) and donor loss (*n* = 15, 26.3%) events, followed by donor gain (*n* = 15, 26.3%) and acceptor gain (*n* = 10, 17.5%) (Fig. 4G). Enrichment analysis for splicing features, RNA-binding protein sites, and other genomic features demonstrated sQTL-SNPs were significantly enriched in many features, and enrichment for splicing sites and RBPs was also high for the 57 predicted causal variants (while intergenic was no longer enriched) (Additional file 2: Fig. S14).

Examining putative causal variants that did not fall within 95% CS revealed some examples where the putative causal SNP is quite far from the sJunction and there are many SNPs in the region showing stronger association (Additional file 2: Fig. S15A). However, other examples, such as rs2302252, showed a strong statistical association and were located close to the sJunction (+ 133 bp) but were not in the 95% CS (Additional file 2: Fig. S15B). The evidence is strongest when the statistical fine-mapping and AI causal variant prediction agree. In addition to the *U2AF1L4* and *OAS1* causal sQTL-SNPs described above, another example of this is rs2297616 in the *PARP2* gene (Fig. 4H).

### Experimental validation of a predicted causal variant: rs2297616 creates a new 5’ splice donor site resulting in a longer protein isoform of PARP2

**Fig. 5:**
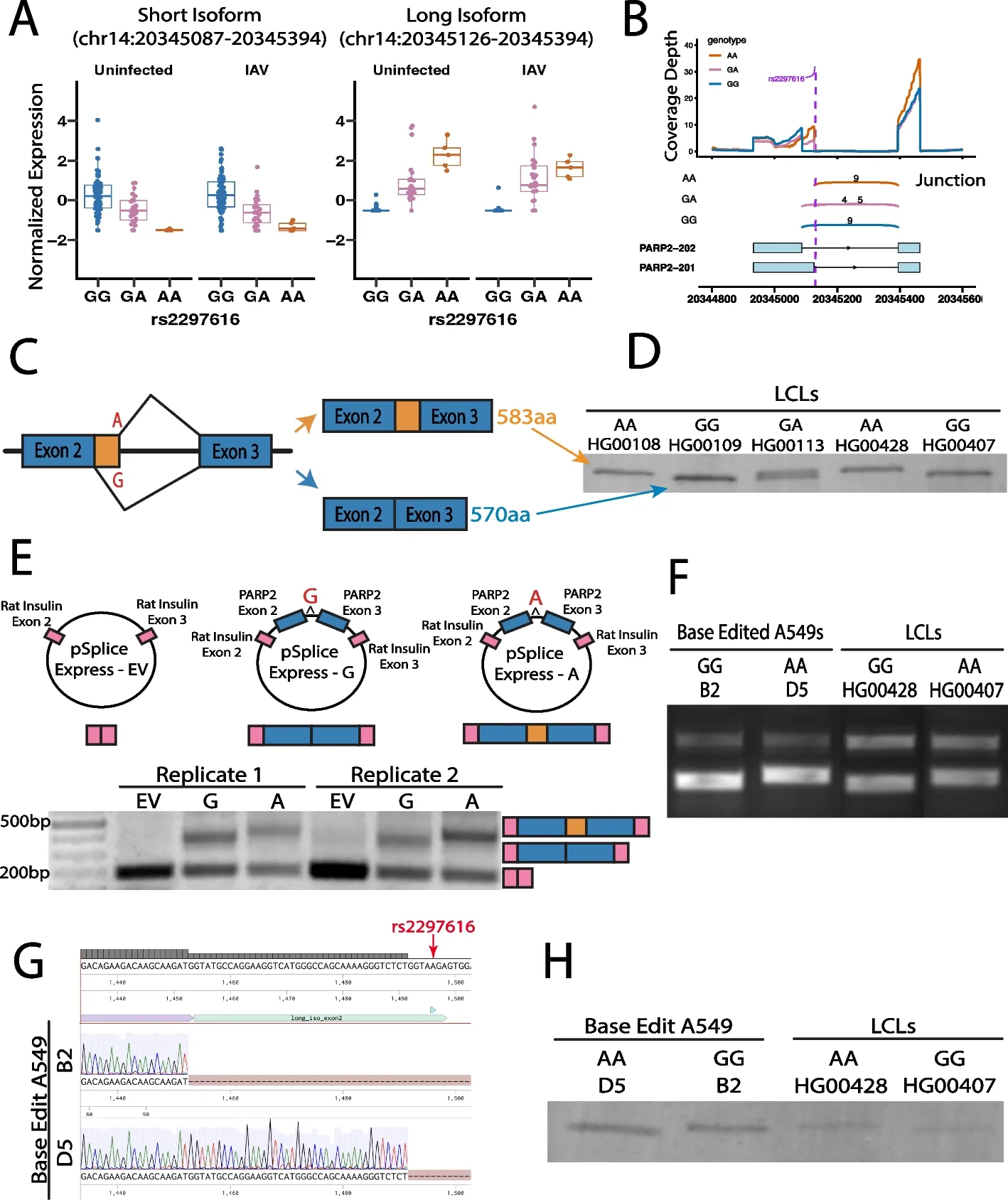
rs2297616 control of 5’ splice donor usage of *PARP2* results in two different protein isoforms. **A** Genotypic boxplots of normalized *PARP2* junction reads for long and short isoforms with or without IAV infection. Boxplots show median and interquartile range, with each dot representing normalized junction reads of a single LCL donor. **B** Sashimi plot of *PARP2* from pseudo-bulk data stratified by rs2297616 genotype. Exon reads are represented by the peaks in the top plot of coverage depth while junction reads are represented by arcs in the middle plot. These reads can be compared to the two *PARP2* isoforms, represented below these plots. **C** Model of predicted splice donor site and protein size based on rs2297616 sQTL data, Sashimi plot, and Maxent scores. **D** Protein lysates from LCLs of either AA (HG00108, HG00507), GG (HG00109, HG00428) or GA (HG00113) genotype reveal PARP2 protein size differences that correlate with genotype. PARP2 protein from AA LCLs migrates slower than PARP2 from GG LCLs, consistent with the A allele causing production of a longer isoform. GA individuals demonstrate two different sized PARP2 proteins. This experiment was conducted 2 times. **E** Agarose gel of cDNA from minigene transfection assay with pSpliceExpress plasmids. pSpliceExpress has nothing inserted between the control rat insulin sequences. pSplice Express-G and pSpliceExpress-A have the DNA sequence from the end of exon 2 to the beginning of exon 3 of *PARP2* inserted with either the rs2297616 G or A allele. Transfection with pSpliceExpress empty vector shows a 200 bp band consistent with the rat insulin exon sequence length. Transfection of pSpliceExpress-G reveals a cDNA product at 425 bp consistent with a shorter splice product. Transfection with pSpliceExpress-A reveals a product at 464 bp which is consistent with a splice donor site and therefore larger splice product. This experiment was conducted 2 times. **F** cDNA from A549 cells that were edited at rs2297616 to have either the G or A allele show splicing consistent with the minigene assay. D5 cell line (homozygous A) have a longer splice product than B2 cells (homozygous G). The cDNA products align with cDNA from LCLs of either genotype. This experiment was conducted 1 time. **G** Sequencing of gel extracted cDNA from (F) shows inclusion of the predicted 39 nucleotides in the AA base edited cells. **H** Protein lysates from base edited A549 cells reveal expected protein isoforms from genotype. D5 (homozygous A) with a larger PARP2 isoform than B2 cells (homozygous G). PARP2 protein isoform size consistent with that of LCL control protein lysates AA (HG00407) and GG (HG00428). This experiment was conducted 3 times

We experimentally validated the mechanism of one of the strongest scoring causal sQTLs, rs2297616, located at the beginning of intron 2 (+ 4 bp) of the *PARP2* gene. This sQTL-SNP was chosen for validation, as in addition to having one of the highest Pangolin AI scores and being in close proximity to the splice junction, it was the only predicted causal sQTL with a nominal association with IAV burden in our scHi-HOST dataset. PARP2, Poly (ADP-ribose) polymerase 2, is a member of the ART enzyme superfamily, which catalyzes post-translational addition of ADP-ribose to proteins in a NAD + dependent manner. There are 17 PARPs in humans, due to paralogous gene duplication, and several have been shown to play a role during viral infections [60]. rs2297616 is associated with two splice junction events in opposite directions. The G allele is associated with greater levels of the chr14:20,345,987–20345394 event and decreased levels of chr14:20,345,126–20345394 (Fig. 5A; Additional file 2: Fig. S16). Examination of splice junctions in a Sashimi plot revealed that the SNP was associated with alternative splicing between exons 2 and 3, appearing to change 5’ splice donor usage (Fig. 5B; as well as in Fig. S16 from AlphaGenome prediction).

The sQTL data support a model where rs2297616 controls which 5’ splice site donor from exon 2 is used (Fig. 5C): If there is a G at rs2297616, an earlier splice site is used, resulting in a predicted protein 570 amino acids in length. However, if there is an A at rs2297616, an alternative splice donor site is created, resulting in a protein 583 amino acids in length. The additional 13 amino acids (amino acids 68–80) are in the disordered N-terminal region of PARP2 and have unknown function. These protein isoforms are the two known common isoforms (Q9UGN5-1 (583 amino acids) and Q9UGN5-2 (570 amino acids), but how their expression is regulated was previously unknown. Maximum entropy modeling supported this predicted splice site change, with the A allele having a greater Maxent score (9.45) than the G allele (6.77), indicative of increased potential for U1 snRNP binding [61].

Western blot of LCLs confirmed that PARP2 isoform size was genotype dependent. LCLs from GG individuals demonstrate a single smaller band than AA, while heterozygous (GA) LCLs reveal two bands of both isoform sizes (Fig. 5D). To determine if the difference in protein size was directly controlled by rs2297616 genotype, we constructed mini-gene splicing reporter plasmids for *PARP2* exons 2–3 with the intervening intron harboring the two distinct alleles (Fig. 5E). PCR of cDNA collected from cells transfected with either splicing reporter revealed different size splice products. The pSpliceExpress plasmid with the A allele revealed a larger band than the pSpliceExpress-G plasmid (Fig. 5E). Further, Sanger sequencing of these bands revealed an additional 39 nucleotides with the A allele—exactly matching the predicted alternative 5’ splice site donor usage (Additional file 2: Fig. S17).

To further investigate how rs2297616 controls splicing and isoform usage of PARP2, we generated CRISPR-Cas9-edited cell lines using homology-directed repair from lung epithelial cells (A549s, which are naturally homozygous G at this locus). Amplification of cDNA from edited cell line clones D5 (homozygous A) and B2 (homozygous G) revealed a difference in cDNA size, similar to that seen with the pSpliceExpress-A and pSpliceExpress-G plasmids (Fig. 5F). Sequencing confirmed the inclusion of the expected 39 nucleotides in the D5 edited cell line, indicating a later splice site was used (Fig. 5G). Finally, these changes in cDNA size resulted in the expected PARP2 protein size difference by western blot (Fig. 5H). These results show that rs2297616 controls the 5’ splice donor usage of exon 2 in *PARP2* to dictate which protein isoform is expressed.

### PARP2 rs2297616 is associated with IAV burden

**Fig. 6:**
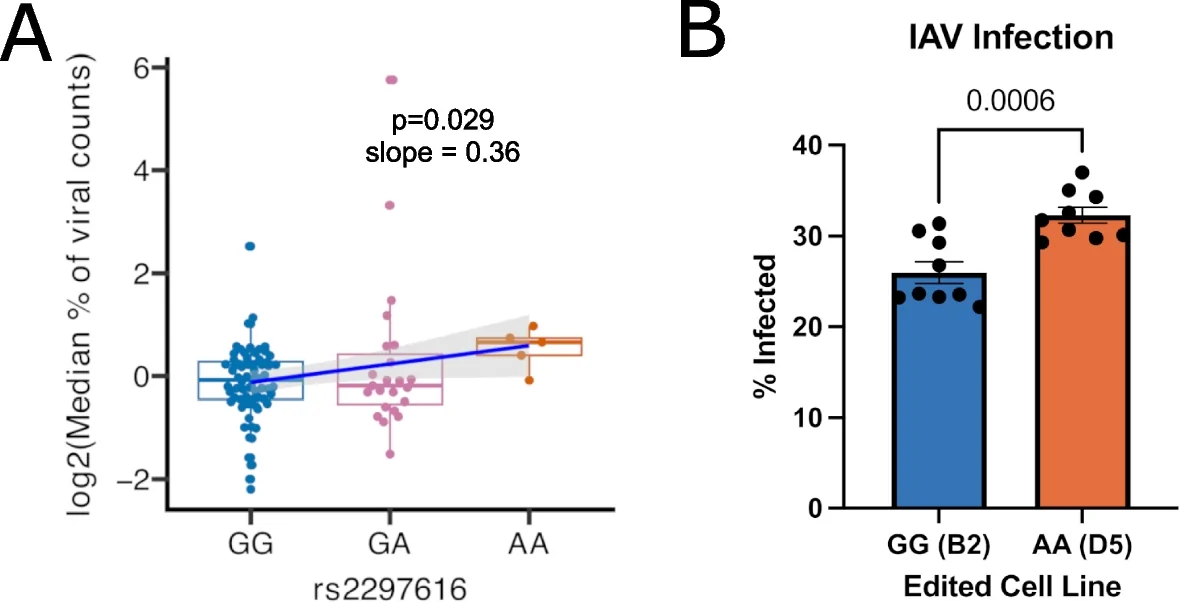
PARP2 rs2297616 A-associated long isoform is more susceptible to CA09 IAV infection. **A** Genotypic boxplot of median percentage of viral counts for rs2297616. The AA genotype is significantly associated with higher median percentage of viral counts. *P*-value is from EMMAX of 96 LCLs. Slope is from linear regression. **B** IAV infection of CRISPR-Cas9-edited A549s read out by flow cytometry confirms A-associated long isoform is more susceptible to IAV infection. Each dot represents a separately plated and infected biological replicates measured across 3 experiments. *P*-value is from an unpaired t-test

Previous studies have implicated PARPs as regulators of IAV infection [62–65]. To investigate whether rs2297616 affects influenza infection, we examined its association with CA09 IAV burden, as measured by median viral count of all single cells for each LCL in our scHi-HOST data. The individuals carrying the rs2297616 AA genotype exhibited modestly higher median viral burden compared to those with GA or GG genotypes (Fig. 6A; *p* = 0.029), suggesting that the A allele (the derived allele causing the longer isoform) likely enhances susceptibility to viral replication.

We tested this hypothesis with our edited A549s. IAV infection of these cell lines showed an effect on viral burden consistent with the sQTL association. The homozygous A edited cell line (D5) showed a significantly higher proportion of infected cells compared to homozygous G cells (B2), further confirming the A allele increases susceptibility to IAV infection (Fig. 6B; *p* = 0.0006).

### scHi-HOST causal sQTLs are associated with diverse human diseases

**Fig. 7:**
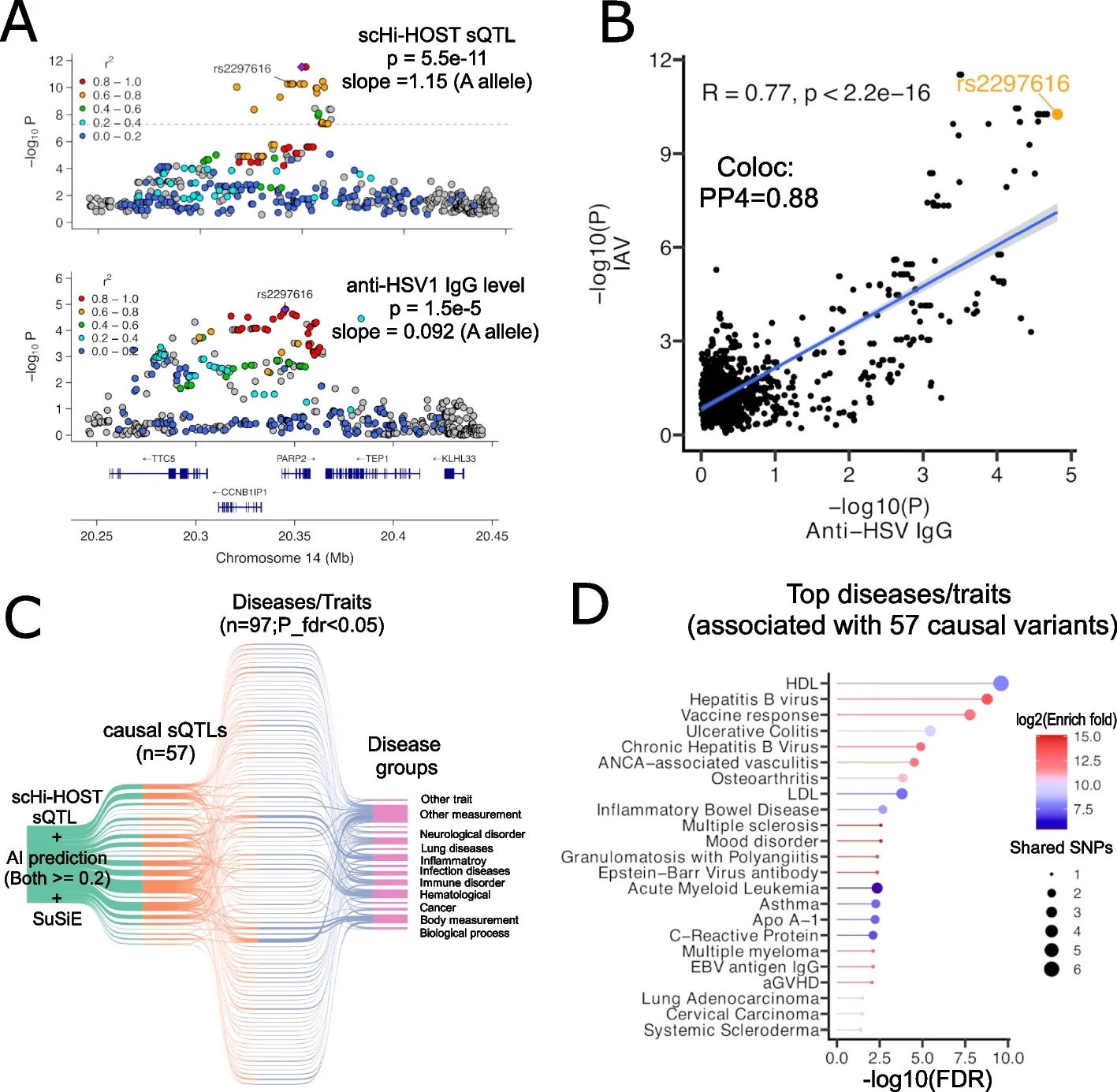
scHi-HOST sQTL is associated with various human diseases. **A** LocusZoom plots of PARP2 sQTL in IAV (this study) and GWAS of anti-HSV1 IgG level. **B** Coloc analysis and *p*-value comparison of PARP2 sQTL and GWAS of anti-HSV1 IgG level. **C** Sankey plot of 57 predicted causal sQTLs based on output of iCPAGdb analysis. **D** Dotplot shows top human diseases that are associated with 57 predicted causal sQTLs. The dot color depicts the significance of *p*-values, and dot size represents the degree of fold enrichment

As noted, none of the sQTLs were associated with patient-reported influenza risk in the largest influenza GWAS, while the *OAS1* and *IFNAR2* sQTL were associated with severe COVID-19 (see Fig. 2E-G and Additional file 2: Fig. S10). To investigate whether rs2297616 impacts viral infections in humans beyond these two GWAS, we searched the previous web browser of OpenGWAS [66] and found a moderate association (*p* = 1.5e-5) with anti-HSV1 (herpes simplex virus 1) IgG levels. We downloaded the full GWAS summary statistics for the study [37] from the NHGRI/EBI GWAS catalog [67]. The A allele, which is associated with higher IAV burden, is also associated with greater risk of HSV1 based on antibody measurement. To test whether the sQTL effect on *PARP2* and the HSV-1 association likely shared a causal variant, we performed colocalization analysis using coloc [9]. The *PARP2* sQTL signal showed strong overlap with the GWAS signal, and rs2297616 was the lead variant for the HSV-1 IgG signal (Fig. 7A). Colocalization was demonstrated by a PP4 of 0.88, and we found significant positive correlation of -log *p*-values SNPs at this locus for the two traits (R = 0.77, *p*-value < 2.2e-16) (Fig. 7B; Additional file 9: Table S8). This shared causality at rs2297616 suggests that alternative splicing of *PARP2* may contribute to interindividual difference in antiviral responses to multiple viral pathogens.

To assess whether other causal sQTL variants were associated with human disease, we performed cross-phenotype analysis for the 57 high-confidence predicted causal sQTLs using our previously developed tool, iCPAGdb [68]. iCPAGdb estimates disease associations based the shared genetic variants using a LD-aware framework and GWAS loci cataloged by the NHGRI-EBI GWAS catalog [67], encompassing variants from more than 4400 diseases. This comprehensive integrative analysis demonstrated that the 57 causal sQTLs were associated more than expected by chance with 97 different diseases/traits (Fig. 7C; Additional file 10: Table S9). Cross-trait analysis revealed these variants are often significantly associated with multiple diseases, including infectious, autoimmune, and metabolic disorders (Fig. 7D). Top associations included hepatitis B virus infection, Epstein–Barr virus infection, asthma, and multiple sclerosis.

Together, these results demonstrate that the integration of single-cell sQTL mapping with AI-based variant prioritization provides a powerful framework for uncovering causal splicing variants underlying complex human diseases.

## Discussion

In this study, we developed a systematic and efficient approach that combines single cell-based sQTL detection with AI-based splice prediction and statistical fine-mapping to predict causal splice variants. Using our scHi-HOST single-cell GWAS platform with uninfected and IAV-infected LCLs, we identified 28,674 sQTL-SNPs from 378 sGenes and localized underlying causal variants to 57 SNPs with deep-learning-based splice prediction that fell within SuSiE 95% CS. These findings also build upon previous studies of the effects of IAV on host splicing [35, 36] and identification of sQTL in the context of IAV infection [33, 34] that did not examine the impact of sQTLs on IAV infection phenotypes or systematic AI-based identification of causal variants as we have now done.

Deep-learning models, such as SpliceAI and Pangolin, leverage sequence information (with Pangolin additionally trained on multi-species data) to infer the effects of variants on canonical and cryptic splice sites, whereas sQTLs quantify how genetic variants are associated with splicing events in populations. The convergence of these complementary signals strengthens causal inference and enables the discovery of common variants that mechanistically alter splicing and contribute to complex disease risk. Only 25 of 57 (43.9%) high-confidence predicted causal variants (both AI ≥ 0.2 and within 95% CS) and 53 of 129 (41.1%) less stringent predicted causal variants (either AI ≥ 0.2 and within 95% CS) were also the variant with the largest PIP for the sQTL signal. Further, as molQTL and GWAS lead variants are often in strong LD with many other SNPs, the integration of QTL, AI, and statistical fine mapping demonstrated here is complementary to colocalization approaches in identifying causal variants that underlie molQTL and GWAS signals and revealing precise mechanisms of action.

The current deep learning-based AI models for splicing have mainly been applied to predicting the impact of rare, splice-disrupting variants [27, 28]. AI has been quite successful in this application, achieving up to 90% sensitivity [55] and being incorporated into clinical genetic workflows [27]. Using deep learning to systematically discover *common* genetic causal variants influencing alternative splicing has received less attention. We found that 26% of sJunctions were assigned a predicted causal variant by at least one of the splicing AI tools that also falls within a SuSiE 95% CS. On average, these predicted causal variants are associated with splicing differences of larger effect size, consistent with the idea these deep learning tools are better at identifying more severe changes that are perhaps under less evolutionary constraint. However, this hypothesis needs further systematic evaluation in future work.

We experimentally validated the mechanistic effect of rs2297616 on *PARP2*, which ranked 5th in splice score prediction. Our analysis and experiments defined how the derived A allele creates a 5’ donor site at the end of exon 2 which lengthens the transcript by 39 nucleotides, therefore creating a PARP2 protein isoform 13 amino acids greater in length. While earlier studies linked rs2297616 genotype to alternative *PARP2* splicing using splicing-sensitive microarray data in HapMap CEU population [69, 70], we have provided the first evidence that connects rs2297616 genotype to differing PARP2 protein isoform expression and used CRISPR editing to stringently assess the effect of this variant in its normal genomic context. Furthermore, we have gone on to connect PARP2 protein isoform expression to IAV susceptibility. Together, these experiments elucidate mechanism from genotype to splice site alteration to protein isoform to viral replication. Our data demonstrates that combining sQTL, AI splicing prediction, and cellular genetic association in scHi-HOST can provide a direct mechanism by which a single nucleotide modulates susceptibility to infection.

PARPs have long been implicated in multiple viral infections, with both reported pro-viral and anti-viral roles. PARP11 and PARP12 have been shown to play a synergistic anti-viral role against Zika virus [71], while PARP12 and PARP14 increase IFN production during mouse hepatitis virus infection [72]. Poly(ADP-ribosyl)ation is induced upon HSV-1 infection and blocked by PARP1/2 inhibition [73]. During IAV infection, PARP1 has been shown by multiple studies to enhance IAV replication [63, 64, 74]. In contrast, PARP13 has been revealed as an antiviral factor against IAV [75] and a recent preprint has shown that all PARPs exhibit antiviral activity against IAV when overexpressed [65]. Since overexpression studies usually examine a single protein isoform, our findings highlight the importance of evaluating isoform usage in infection outcomes, especially in regard to PARP proteins as previous studies have suggested [76]. For PARP2, this extends beyond IAV infection as we have found that the same sQTL locus colocalized with anti-HSV-1 IgG, indicating that these two PARP2 isoforms have broader roles during viral infection that will be investigated mechanistically in future work.

Our study has limitations. First, the dataset used in this study includes only 96 samples from six global populations, which may limit power for discovering sQTLs of more modest effect and decreases resolution in fine mapping. Expanding to more diverse ancestry samples and additional cell types will enhance sQTL discovery by inclusion of lower frequency variants and cell-type specific splicing junctions. Second, the junction readouts are based on short read sequencing which rely on algorithmic calling. Long-read sequencing would improve sQTL mapping by directly quantifying full-length transcript isoforms and reducing the error of junction readouts in complex sequence regions, such as segmental duplications. Third, the analysis we have conducted utilized only pseudobulk analysis. In future studies, power would likely be improved by incorporating heterogeneity from the scRNA-seq into the sQTL pipeline (e.g. as in [77]), while additional rsQTL and isQTL may be identified in examining sub-populations of cells.

## Conclusions

Our study provides 1) a catalog of predicted causal cis-sQTLs with putative mechanism of action, 2) experimental validation of an sQTL that changes PARP2 protein length to regulate IAV infection in cells, and 3) a generalizable framework for moving from association to mechanism for complex traits. These insights highlight how combining sQTL mapping, AI, fine mapping, and functional validation can enhance discovery of disease causal variants with a clear genetic mechanism of differential splicing, enabling possible therapeutic and diagnostic strategies.

## Methods

### Cell lines

Lymphoblastoid cell lines (LCLs; EBV-immortalized B cells) from the 1000 Genomes Project were purchased from the Coriell Institute. LCLs from 6 populations were included, LWK (Luhya in Webuye, Kenya), ESN (Esan in Nigeria), GBR (British in England and Scotland), IBS (Iberian in Spain), CHS (Han Chinese South), and KHV (Kinh in Ho Chi Minh City, Vietnam). LCLs were maintained at 37 °C in a 5% CO_2_ atmosphere and were grown in RPMI 1640 media (Gibco #21,870) supplemented with 10% heat-inactivated fetal bovine serum (HI-FBS, Gibco #10,082), 2 mM L-glutamine (Gibco #25,030,081), and 100 U/ml Penicillin-G, and 100 mg/mL Streptomycin (Gibco #15,140,122). Genetic demultiplexing successfully recovered all 96 cell lines, authenticating the presence of these specific LCLs in the scHi-HOST dataset. A549 cells (ATCC) were grown in DMEM + 10% FBS + 1% Pen-Strep. Cells were periodically tested for mycoplasma contamination and none was detected.

### Influenza A virus infections

The CA09 (California/7/09, H1N1) sfGFP-HA strain [78] was propagated in MDCK cells and used to infect LCLs and A549 cells. Briefly, virus was used to infect MDCK cells at a low MOI in Opti-MEM-based media containing TPCK-treated trypsin, and nascent virus containing supernatant was collected 48–72 h post-initial infection [79]. To increase the number of infectious units per volume, virions were concentrated prior to use via centrifugation over a sucrose cushion as previously described [80].

For scHi-HOST, LCLs were infected in two groups of 48 LCLs, scHi-HOST Neo (previously scHH-EIK in [29]) and scHi-HOST Morpheus (previously scHH-LGC in [29]). LCLs were pooled in equal number and 250,000 total cells were plated in each well of a 24-well plate in PBS with 0.35% BSA, 2 mM glutamine, 100 U/mL penicillin-G, and 100 mg/mL streptomycin. Cells were infected at MOI 25 or left as uninfected controls. At 3 h post infection, each well was spiked with 600 μL of RPMI 1640 media (Invitrogen) supplemented with 10% fetal bovine serum (FBS), 2 mM glutamine, 100 U/mL penicillin-G, and 100 mg/mL streptomycin. At 24 h post infection, cells from each sample were collected, spun down, and resuspended in PBS with 0.04% BSA for single-cell cDNA library preparation.

For A549 infection experiments, A549 cells were plated at 20,000 cells per well in 100 μL growth media (DMEM + 10% FBS + 1% Pen-Strep) in TC-treated 96 well plates. 1 day after plating, media was removed and replaced with 50 μL PBS with 0.35% BSA, 2 mM glutamine, 100 U/mL penicillin-G, and 100 mg/mL streptomycin. Cells were then infected with an MOI of 10. 1 hpi, media was removed and replaced with 50 μL of Opti-mem + 0.01% FBS + 1% Pen-Strep + 0.35% BSA. 24 hpi, cells were trypsinized and assayed for percent GFP + using a Guava Easycyte flow cytometer.

### Single cell RNA-seq library preparation, sequencing, and alignment

LCLs were counted using a Guava EasyCyte HT with 7-AAD staining. The scRNA-seq cDNA library was barcoded and prepared using the 10 × Chromium Single Cell 3’ Library and Gel Bead Kits v3.1 (Pleasanton, CA). The scRNA-sequencing resulted in 31,155 and 13,794 cells for uninfected (scHi-HOST NeoUninfected 11,180 + scHi-HOST MorpheusUninfected 19,975) and CA09 IAV (scHi-HOST NeoCA09 8861 + scHi-HOST MorpheusCA09 4933).

cDNAs were sequenced on an Illumina NovaSeq S4 flow cell, with a targeting depth of 100,000 reads per barcoded droplet for infected library. The details of sequencing information for uninfected can be found in our previous publication [29].

Paired-end short reads were mapped to a custom reference genome using *CellRanger* v7.0.1 [81] with default parameters. The custom reference genome combined human GRCh38 reference genome and influenza A/California/7/09 reference genome. Sequencing was conducted in two batches, with each of 48 LCLs. For scHi-HOST Neo, the library was single-indexed, and index-hopped reads were removed using 10 × Genomics Index-hopping-filter software (https://github.com/10XGenomics/index_hopping_filter) before aligning to the custom reference genome. For scHi-HOST Morpheus, the library was dual-indexed. Reads were sequenced with read 1 length of 28 base pairs (bp) and read 2 length of 150 bp.

Each droplet was assigned to individual LCL identity using Demuxlet [31]. Demuxlet estimates the likelihood of each droplet identity based on natural genetic variations, matching the SNPs from mRNA reads with individual LCL genotypes. The input bam file of Demuxlet was directly taken from *CellRanger* output. The other input file included a VCF file that contained genotypes for 96. We used Subset-Bam software (10X Genomics) to extract reads from each CellRanger alignment bam into 96 pseudobulk bam files. Pseudobulk bam files were further used for sQTL detection, viral burden quantification, and other analysis.

### LCLs genotypes

LCL genotypes were obtained from the high coverage whole genome sequencing (30x) of 1000 genome project phase 3, generated by New York Genome Center (NYGC; download link: https://www.internationalgenome.org/data-portal/data-collection/30x-grch38). The bcftools v1.9 [82] was used to prepare the VCF genotype file and then filtered the VCF file with parameters of “-q 0.05:minor -m 2 -M 2”, to restrict our analysis to only biallelic SNPs and those with minor allele frequency more than 0.05. Genotypic principal components were calculated using Plink v1.9 [83].

### Identification of splicing QTLs (sQTLs)

We performed splicing QTL (sQTL) analysis using an annotation-free pipeline, *Leafcutter* v0.2.9 [16], adapted from the GTEx v8 pipeline (https://github.com/broadinstitute/gtex-pipeline). Transcript junction annotations were preprocessed by collapsing isoforms per gene using collapse_annotation.py based on the GTF file from GENCODE v32. RegTools (v0.5.1) [84] was employed to count junction reads for each LCL based on pseudo-bulk BAM file from CellRanger output. The following default parameters were used by RegTools: minimum anchor length of 8 bp (“-a 8”), intron size between 50 bp and 500,000 bp (“-m 50 -M 500,000”), and unstranded mode (“-s 0”). Leafcutter “leafcutter_cluster_regtools.py “ was used for intron clustering for extracted splice junctions, with parameters of filtering out clusters with < 30 split reads (“–min_clu_reads 30”), the minimum fraction of reads supporting junctions in a cluster of 0.001 (“–min_clu_ratio 0.001”), and maximum intron length of 500,000 (“–max_intron_len 500,000”). Leafcutter “prepare_phenotype_table.py” was used to convert intron reads to splice junction ratio and perform quantile normalization. To capture latent confounding factors underlying the junction ratio matrix, we calculated probabilistic estimation of expression residuals (PEER) factors using R peer v1.3 [85] based on the normalized junction ratio matrix.

To assess the impact of read depth on sQTL detection, we binned genes by expression level and computed the probability of detecting ≥ 1 sQTL (FDR < 0.05). The probability of detecting a sQTL was defined as the fraction of genes in each bin associated with at least 1 sQTL. This probability rose with expression, peaking at ~ 10,000 reads (Additional file 2: Fig. S6). Mean read depth per gene was 366 (uninfected) and 283 (IAV), defined as 1x. At 1x, sQTL detection probability was ~ 2.5%, dropping to 0.4% at 0.1 × and rising to ~ 7% at 10x, with a plateau near 10x. This indicates that higher read depth enhances sQTL detection.

We then used TensorQTL (v1.0.6) [86] for sQTL mapping based on the phenotype bed files generated from Leafcutter. The SNPs located within 100,000 bp window of a gene’s starting and/or end position were included for the SNP-junction association testing. To account for potential cofounding factors, the first two genotypic principal components (PCs), sex, and top 3 PEER factors were incorporated in the regression model. As some PEER factors were correlated (Additional file 2: Fig. S18A, B), we evaluated pairwise correlations among the top 10 PEER factors using the R “cor” function. With a *p*-value cutoff of 0.05, we retained the top three PEER factors. The remaining seven PEER factors were correlated with these top three factors in both uninfected and IAV conditions. We also benchmarked sQTL detection using sex, top two genotypic PCs as covariates, together with varying numbers of PEER factors (*n* = 2, 3, 5, 10, 15) (Additional file 2: Fig. S18C-H).

For sQTL analysis, nominal *p*-values were computed for all SNP-junction pairs from TensorQTL [86]. For each junction, empirical *p*-value for the most significant SNP per junction was calculated under the β distribution approximation using the adaptive permutations with parameters of “–permute 1000 10,000”. The Benjamini–Hochberg false discovery rate (FDR) method was applied to identify significant splicing genes (sGenes) at an FDR level of 0.05. The significance of sQTL per sGene was defined by comparing their nominal *p* value to the nominal *p*-value threshold of the matching sGene.

### IAV response sQTL

To identify IAV response sQTL, we performed a linear mixed-effects model (LMM) analysis. The quantile-normalized junction usage was modeled as a function of genotype, treatment, covariates, and donor as a random effect, and rsQTL were assessed by testing the genotype-by-treatment interaction using a likelihood ratio test (LRT). Specifically, for each candidate sQTL SNP-junction pair, we modeled quantile normalized junction usage (y) as a function of the SNP genotype, treatment (0 for uninfected, 1 for IAV stimuli), the top two genotypic PCs, sex, and the top three PEER factors, with donor (i.e. LCL sample) as a random effect in both models. The initial equations M0 were used as follows:

$$\mathrm{M}0: \mathrm{y}\;\sim\;\mathrm{SNP}\_\mathrm{geno}\;+\;\mathrm{treatment}\;+\;\mathrm{gPC}1\;+\;\mathrm{gPC}2\;+\;\mathrm{sex}\;+\;\mathrm{PEER}1\;+\;\mathrm{PEER}2\;+\;\mathrm{PEER}3\;+\;(1\;|\;\mathrm{donor})$$

We then fit a second model (M1) with similar settings as M0 but including the genotype*treatment interaction term:

$$\mathrm{M}1:\;\mathrm{y}\;\sim\;\mathrm{SNP}\_\mathrm{geno}\;+\;\mathrm{treatment}\;+\;\mathrm{gPC}1\;+\;\mathrm{gPC}2\;+\;\mathrm{sex}\;+\;\mathrm{PEER}1\;+\;\mathrm{PEER}2\;+\;\mathrm{PEER}3\;+\;\mathrm{SNP}\_\mathrm{geno}:\mathrm{treatment}\;+\;(1\;|\;\mathrm{donor}).$$

We then used a likelihood ratio test (LRT) to compare M0 and M1 and obtained the *p* values from interaction term. The significance of rsQTL was defined as those with q-value < 0.1.

We grouped sQTLs into three categories: response sQTL (rsQTL), constitutive sQTL (csQTL), and induced sQTL (isQTL). All sQTL SNP-junction pairs that were detected under the IAV infection condition were classified as rsQTL (q-value < 0.1) or csQTL (q-value > 0.1). sQTL SNP-junction pairs that were detected only under the IAV infection condition (not detected in uninfected or extremely low and filtered out by Leafcutter’s default filtering parameter (“–mincluratio 0.001” which indicates the minimum fraction of reads in a cluster that support a junction)) were classified as isQTL.

As a more relaxed post-hoc method, we also applied the z-test approach to define IAV response sQTL (rsQTL) as described in previous work [87, 88]. The z-test compares the slope difference of a sQTL SNP-junction pair between uninfected and IAV conditions, where the slope values were extracted from TensorQTL output. The equation we used was as follows:

$$\mathrm{z}=\frac{{\mathrm{slope}}_{\mathrm{I}\mathrm{A}\mathrm{V}}-{\mathrm{slope}}_{\mathrm{u}\mathrm{n}\mathrm{i}\mathrm{n}\mathrm{f}\mathrm{e}\mathrm{c}\mathrm{t}\mathrm{e}\mathrm{d}}}{\sqrt{\mathrm{s}\mathrm{l}\mathrm{o}\mathrm{p}{\mathrm{e}\_\mathrm{s}\mathrm{e}}_{\mathrm{I}\mathrm{A}\mathrm{V}}+{ \mathrm{s}\mathrm{l}\mathrm{o}\mathrm{p}\mathrm{e}\_\mathrm{s}\mathrm{e}}_{\mathrm{u}\mathrm{n}\mathrm{i}\mathrm{n}\mathrm{f}\mathrm{e}\mathrm{c}\mathrm{t}\mathrm{e}\mathrm{d}}} }$$

The resulting *p*-value were further corrected using Benjamini–Hochberg method from R “p.adjust” function.

### Differential splicing genes (DSGs)

Differential splicing analysis was tested using the Leafcutter’s *leafcutter_ds.R* function, incorporating sex as a covariate. Leafcutter employs a likelihood ratio test to compare differences in intron usage between IAV-infected LCLs and uninfected controls. Junctions with a false discovery rate (FDR) ≤ 0.05 were considered significant. The significant junctions were subsequently annotated to their associated genes to facilitate functional enrichment and downstream analysis.

### Annotation of alternative splicing event

The R *psichomics* package [89] was employed to annotate the Leafcutter junction. The hg38 genomic coordinates of these junctions were extracted as input. The resulting annotation includes skipped exon (SE), mutually exclusive exons (MXE), alternative first exon (AFE), alternative last exon (ALE), alternative 5’ splice site (A5SS), alternative 3’ splice site (A3SS).

### sQTL replication in GTEx

We used π_1_ statistic [90] to estimate sQTL enrichment in GTEx sQTL datasets for all 49 tissues (v8). For each SNP-junction pair from scHi-HOST, the nominal *p*-value was extracted from the matched pair in GTEx. Then we used the R *qvalue* package to compute π_0_, and the fraction of true replicated sQTLs or enrichment degree π_1_ is defined as 1- π_0_. As some SNP-junction pairs are not tested or missing in GTEx sQTL, a random *p* value was assigned from uniform distribution.

### Functional enrichment analysis of sQTL variants

To evaluate the global functional context of the sQTLs, we compiled annotation sets relevant to splicing and gene regulation. These included RNA-binding protein (RBP) binding sites downloaded from ENCODE eCLIP datasets (combined K562 and HepG2), candidate cis-regulatory elements (cCREs) from ENCODE SCREEN, chromatin-state annotations from the Roadmap Epigenomics Project, and genic annotations (GENCODE v47), including splice sites with ± 2 bp and ± 10 bp window, exon, etc. We performed enrichment analysis using GREGOR [91], which evaluates overlap of input variants with pre-defined functional annotations relative to matched control SNP sets. European population of the 1000 Genomes Project was used to calculate LD among SNPs. Control SNPs were matched on minor allele frequency, LD, and distance to the nearest gene. Enrichment was quantified as observed versus expected overlap, and statistical significance was assessed by comparison to the matched background sets. The Benjamini–Hochberg method was used to correct *p* values from multiple comparisons.

### Genetic association for flu burden

Short reads were mapped to the influenza A/California/07/2009(H1N1) genome (GenBank accession: GCA_001343785.1) using CellRanger v7. To remove possible ambient RNA contamination of IAV reads, we used CellBender v.0.3.0 [92] with “–fpr 0.01” and “–epochs 150". CellBender uses a deep generative model to learn a background noise profile to delineate cell-containing and empty droplets and provide noise-free gene count quantification. To identify IAV burden phenotypes, the percentage of viral reads to total reads per cell was calculated. The median viral percentage of reads across all cells per LCL was then used as a pseudobulk phenotype. Genetic association testing was performed using EMMAX [93], which uses a linear mixed model and accounts for population stratification and cryptic relatedness by incorporating pairwise kinship across samples. The kinship matrix was calculated based on autosomal SNPs with minor allele frequency (MAF) > 5%. We incorporated biological sex as a covariate to account for possible sex differences.

### Variant functional annotation

We used Ensembl variant effect predictor (VEP v106) [94] to annotate all genomics variant SNPs using parameters “–everything –offline –check-existing.” Broadly, variants are grouped by their relative location into the following types: introns, exons, 3’ UTR, 5’ UTR, and intergenic, or by functions, such as, missense, synonymous mutation, frame shift, splicing donor or acceptor, or splicing region (1–3 bp of exon or 3–8 bp of the intron) etc.

### Transcript quantification

To quantify abundance of individual transcripts per gene, we re-mapped short reads for each LCL to GRCh38 using *Salmon* [95]. The transcript abundance was imported and normalized using R *tximport* [96] and *DESeq2* [97] package. Raw counts were log-transformed with the addition of one pseudo-count.

Transcript-level read counts for IFITM1-201 and IFITM1-202 were quantified using *Salmon* [95] and imported into R with *tximport* [96]. Individual raw count between IFITM1-201 and IFITM1-202 were computed, and paired Student’s t-tests were used to evaluate statistical significance between uninfected and IAV.

### AI-based splicing prediction

We applied both SpliceAI [23] and Pangolin [25] to predict the splicing impact of genetic variants and to annotate the sQTLs. For SpliceAI, we downloaded the pre-computed “genome_scores_v1.3” table from the Illumina BaseSpace platform (https://basespace.illumina.com/). The pre-computed tables provide genome-wide delta scores (DS) and delta distance for all possible SNV and indels based on GRCh38 reference, representing the predicted potential that a variant alters acceptor or donor splice sites [23].

For the Pangolin analysis, we installed Pangolin v1.0.2 locally and ran predictions using a ± 100 bp window around each variant (i.e., the “-d 100” option). For each individual variant, Pangolin estimates the maximum predicted gain and maximum predicted loss in splice-site usage within the given window. 

For both Pangolin and SpliceAI we used the “delta score”, defined as the difference between reference and variant allele predictions, as the key indicator of splicing. A higher delta score implies a greater likelihood of a splice-impacting event. As suggested by the Pangolin authors [25], we adopted a delta score > 0.2 from either tool as a robust indicator that the variant may alter splicing.

To further evaluate the splicing effects of sQTLs in independent datasets, we utilized the recently released AlphaGenome software (v0.4) [56]. AlphaGenome implements the S2F (Sequence-to-Function) deep learning model, which is optimized for predicting the molecular consequences of genetic variants on transcriptome-level features with integration of large-scale cohorts of RNA-seq datasets. All predictions were performed using the human GRCh38 reference genome and gene annotation of GENCODE v46. Two ontology terms, EFO:0000572 and EFO:0008952, were used in the analysis, matching to GTEx EBV-transformed LCL and Lung. For each variant, AlphaGenome extracted the surrounding 1 Mbp genomic sequence and generated predictions for both the reference and alternate alleles. The model provides estimates across multiple publicly available RNA-seq datasets (e.g. GTEx), including predicted splicing effects, splice-site usage, and splice-junction counts. The splice effect of variant and splice junction were visualized using AlphaGenome “plot_components” module.

### Statistical fine mapping with SuSiE

We performed fine-mapping for each sQTL locus using SuSiE [57] based on individual-level genotype and splice junction. Briefly, for each junction, we extracted all variants within 100,000 bp window of the tested locus and constructed a genotype matrix for samples with non-missing phenotype and covariate data. Splicing junction phenotypes were obtained from Leafcutter and adjusted with the same covariates used in the sQTL analysis, including top 2 genotype PC, sex, and top 3 PEER factors. SuSiE was run on normalized junction phenotype using default settings, with a maximum of 10 credible sets (CS) per locus. For each variant, we extracted the posterior inclusion probability (PIP) and identified 95% credible sets as the minimal set of variants whose cumulative posterior probability reached 0.95. Variants with higher PIP were considered more strongly supported as putative causal candidates. We also ran function-informed SuSiE fine-mapping by using AI splice score as prior weights.

### Connecting causal sQTL to human diseases/traits

To explore potential shared causal genetic effects between sQTLs and public GWAS signals, we performed two independent approaches. First, we performed colocalization analysis using the R package *coloc* [9]. The *coloc* package implements a Bayesian framework to assess the probability of colocalization by integrating summary statistics from QTL and GWAS datasets. We applied default prior parameters (p_1_ = 1 × 10⁻^4^, p_2_ = 1 × 10⁻^4^, and p_12_ = 1 × 10⁻^4^) during the analysis. Evidence of colocalization was defined as a posterior probability of colocalization (PP4) greater than 0.8, indicating a high likelihood of shared causal variants between the sQTL and GWAS signals. LocusZoom visualization for *coloc* results were plotted using the *Locuszoomr* R package [98].

As an alternative approach, we performed a systematic cross-phenotype analysis using our previous developed tool, iCPAGdb [68]. This tool connects user’s SNP set to over 4400 traits from NHGRI/EBI GWAS Catalog using a LD-aware approach. European from the 1000 Genome project was chosen for LD reference. The *p*-values were corrected from multiple test correction using R “p.adjust” method.

### Immunoblotting

Cells were lysed with RIPA lysis buffer: 50 mM Tris–HCl pH 7.4, 150 mM NaCl, 0.1% SDS, 0.5% NaDeoxycholate, 1% Triton X-100, cOmplete Mini protease inhibitor cocktail (Millipore Sigma #11,836,170,001), 10 mM NaF, and 1-mM Na orthovanadate. SDS-PAGE was performed using Mini-PROTEAN TGX Stain-Free Precast 4%–20% gels (Bio-Rad #456–8096). The gels’ stain-free dye was activated by a 5-min UV exposure and protein was transferred to Immun-Blot low-fluorescence PVDF membrane (Bio-Rad #162–0264) using Hoefer TE77X. Blots were developed using LI-COR infrared secondary antibodies (IRDye 800CW Donkey anti-rabbit IgG) and imaged on an LI-COR Odyssey Classic. Primary antibodies used: anti-PARP2 pAb (Active Motif RRID: AB_2793328). Uncropped gel images are provided in Additional file 2: Fig. S19.

### Minigene cloning and transfection

The backbone minigene plasmid, pSpliceExpress, was a gift from Stefan Stamm (Addgene plasmid # 32,485; http://n2t.net/addgene:32485; RRID:Addgene_32485) [99]. DNA was extracted from LCL HG00428 and PCR was performed with primers attb1F and attb2R in Additional file 2: Table S10 and Phusion DNA polymerase (Neb). A Gateway BP clonase II reaction (Thermo Fisher) was then preformed overnight at 25C. Site-directed mutagenesis was carried out using Quikchange II XL Site-Directed Mutagenesis Kit (Agilent, #200,521) (primers in Additional file 2: Table S10). A549 cells were seeded at 1.05 × 10^5^ cells per well in a 24 well TC treated plate 24 h before transfection. Transfection was accomplished with Lipofectamine 3000 (Thermo Fisher, Catalog #L3000008). Cells were harvested 48 h post transfection.

### Cas9-RNP editing of A549s

To assemble RNP complexes, we utilized a previously described method [100], with the following modifications for A549 cell type and rs2297616. Three gRNAs for rs2297616 were designed based on GGC site proximity (Additional file 2: Table S10). Centrifugation was performed at 400 × g for 5 min. Both homologous DNA repair fragments (IDT, Alt-R Donor Oligo, 2 nmol) were added to each RNP tube at a concentration of 2.5 μM each from a 20 μM stock (Additional file 2: Table S10). Each reaction was pulsed twice at 1200 V for 30 ms. Cells were then transferred into 500 μL of DMEM + 10% FBS media to incubate for 48 h at 37C. Cells were then split into a TC-treated 6-well plate and DNA was extracted with QuickExtract (Bioresearch Technologies, QE0905T) from each pool. PCR for confirmation of SNP edit was performed using DNA polymerase Phusion (New England Biosciences, M0530S) with primers rs2297616_F and rs2297616_R (Additional file 2: Table S10) at the following cycle conditions: 1X 98 °C 30 s, 30X 98 °C 10 s, 70 °C 45 s, 72 °C 30 s, 1X 72 °C 10 min. PCR product was sent for sanger sequencing (Azenta) with primer: rs2297616_F (Additional file 2: Table S10) and edit frequency for each pool was evaluated by ICE Analysis (EditCo Bio Inc.). Pool from gRNA1 had the highest edit frequency of 15%. Cells from gRNA1 pool were then plated at a concentration of 0.5 cells per well in TC-treated 96 well plates for clonal expansion. Two weeks post seeding, cells were expanded for DNA collection with QuickExtract (Bioresearch Technologies, QE0905T). PCR product was sent for sanger sequencing (Azenta) with primer: rs2297616_F (Additional file 2: Table S10) and edit was visualized with Benchling (https://benchling.com).

### RNA extraction, cDNA synthesis and gel electrophoresis

RNA was extracted using Qiagen kit according to manufacturer’s instructions (Qiagen Cat#74,104). cDNA was made from 500 ng of RNA with iScript (Bio-Rad). PCR products were examined by electrophoresis at 100 V for 30 min in a 2% (w/v) agarose gel with 0.5 μg/mL ethidium bromide in 1 × TAE buffer. Gel extractions were performed with Qiagen kit (Cat#28,706) then analyzed by Sanger sequencing with primer rs2297616_F.

## Supplementary Information

Additional file 1: Table S1. Differential splicing analysis comparing uninfected and IAV-infected conditions

Additional file 2: Fig. S1-S19 and Table S10

Additional file 3: Table S2. Gene set enrichment of differentially spliced genes using Enrichr and the MSigDB hallmark gene set

Additional file 4: Table S3. Significant sQTL along with SuSiE fine mapping for uninfected and IAV-infected conditions

Additional file 5: Table S4. Gene set enrichment of sGenes using Enrichr and the MSigDB hallmark gene set

Additional file 6: Table S5. IAV induced sQTLs and response sQTLs identified by LMM + LRT test and by z-test

Additional file 7: Table S6. AI-predicted causal sQTL-SNPs

Additional file 8: Table S7. SuSiE fine mapping of AI-predicted causal sQTL-SNPs

Additional file 9: Table S8. Colocalization analysis of PARP2 sQTL and anti-HSV-1 IgG

Additional file 10: Table S9. iCPAGdb analysis of causal sQTL-SNP shared with other human traits/diseases in the NHGRI-EBI GWAS Catalog

## Data Availability

The data generated and analyzed during this study are included in this published article and its supplementary information files. The scRNA-seq data for uninfected and CA09-infected 96 LCLs are available from GEO Repository (Accessions GSE205796 [101] and GSE335087 [102]). Analyzed data (differential splicing, enrichment analyses, sQTL identification, fine mapping, AI integrated results, colocalization, and iCPAGdb output) are included as Supplemental Tables (Additional files 1, 3-10; Tables S1-S9). This manuscript used published, publicly available sequencing data of 1000 Genomes LCLs [103], [104]. Code generated for this project is publicly available under the MIT License at Github [105] and Zenodo [106] https://doi.org/10.5281/zenodo.21134762).
