# Supplementary material for: Integrating single-cell sQTL mapping with deep-learning splicing prediction identifies causal variants under influenza infection
Source: Genome Biol. 2026 Jul 18;27:314. doi: 10.1186/s13059-026-04205-9 (PMC13663244; doi:10.1186/s13059-026-04205-9)
Supplement: Supplementary file 1 [file 13059_2026_4205_MOESM2_ESM.pdf]

Supporting Figures and Tables

Fig. S1-S19

Table S10

Fig. S1. Intron junctions identified by Leafcutter classified into 6 types. On the left (blue), all junctions are counted, while the right barplots (red) count junctions that are significantly differentially spliced between IAV-infected and uninfected conditions at qvalue<0.05. SE: skipped exon; AFE, Alternative first exon; A5SS, alternative 5' splice site; A3SS, alternative 3' splice site; MXE, mutually exclusive exon; ALE, alternative last exon.

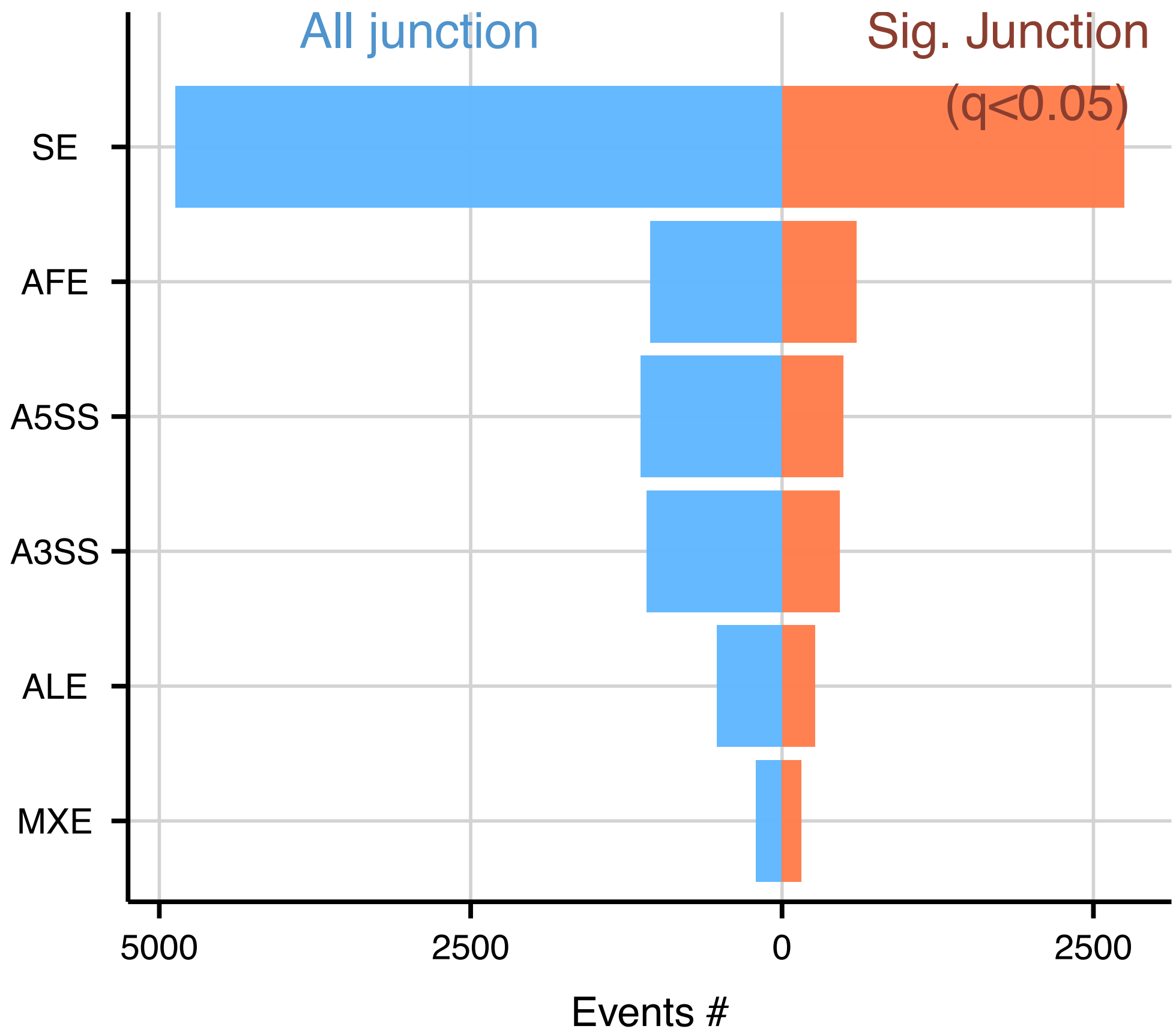

Fig. S2. Barplot of gene set enrichment analysis of genes carrying significant differential junctions between IAV and uninfected from Leafcutter. Enrichment was performed using Enrichr based on MSigDB database. Vertical dashed line indicates FDR of 0.05.

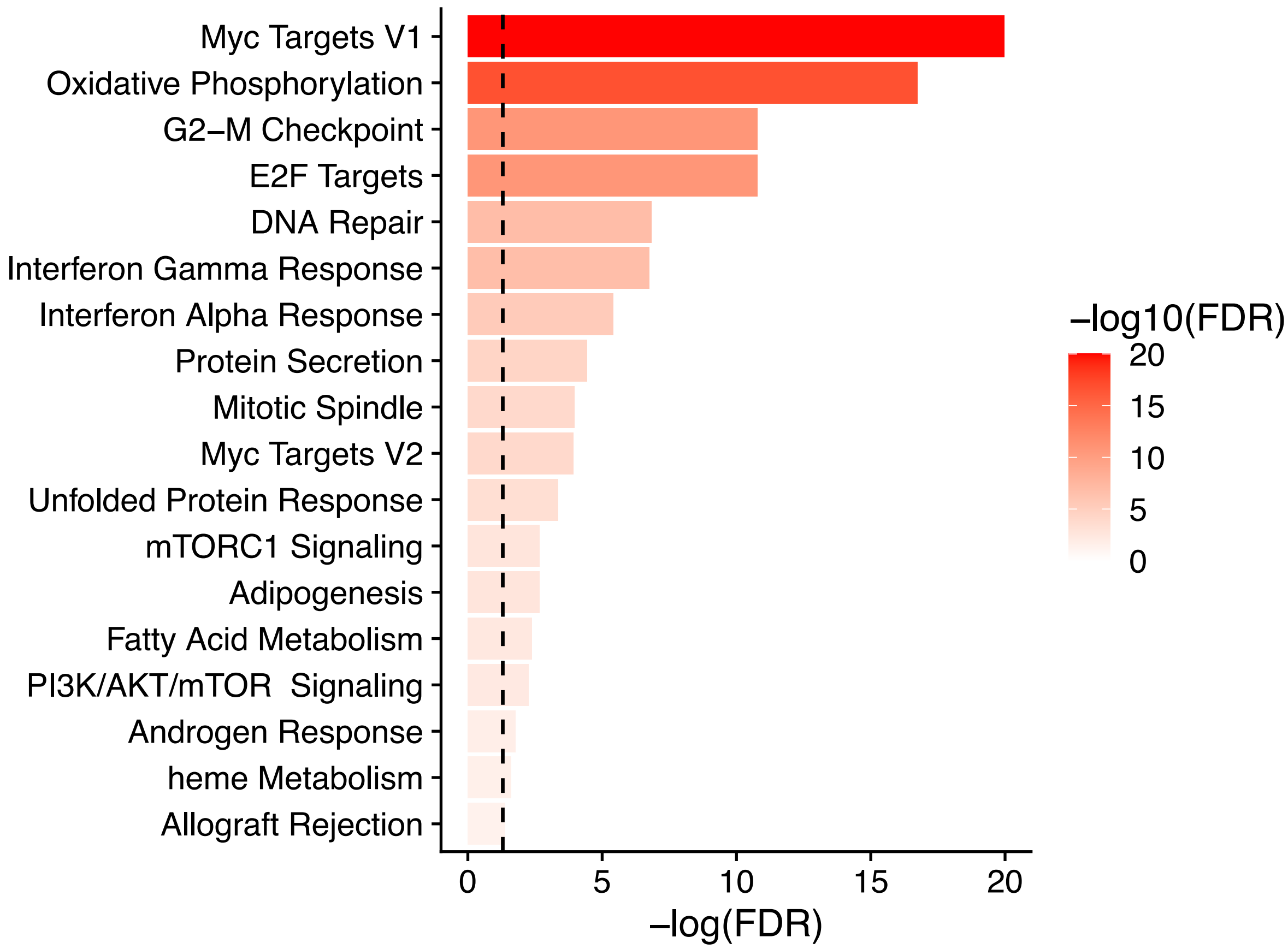

Fig. S3. Single cell visualization of viral burden, interferon genes and pathways. **A)** UMAP of IAV (CA09)-infected and uninfected revealed a total of 12 clusters. **B)** UMAP visualization of percent of viral reads per cell. **C)** UMAP visualization of *IFITM1* gene expression. **D)** Violin plot of *IFITM1* gene expression, and Interferon Alpha and Gamma pathways scores. The Interferon Alpha and Gamma pathways scores were calculated using R Seurat “AddModuleScore”.

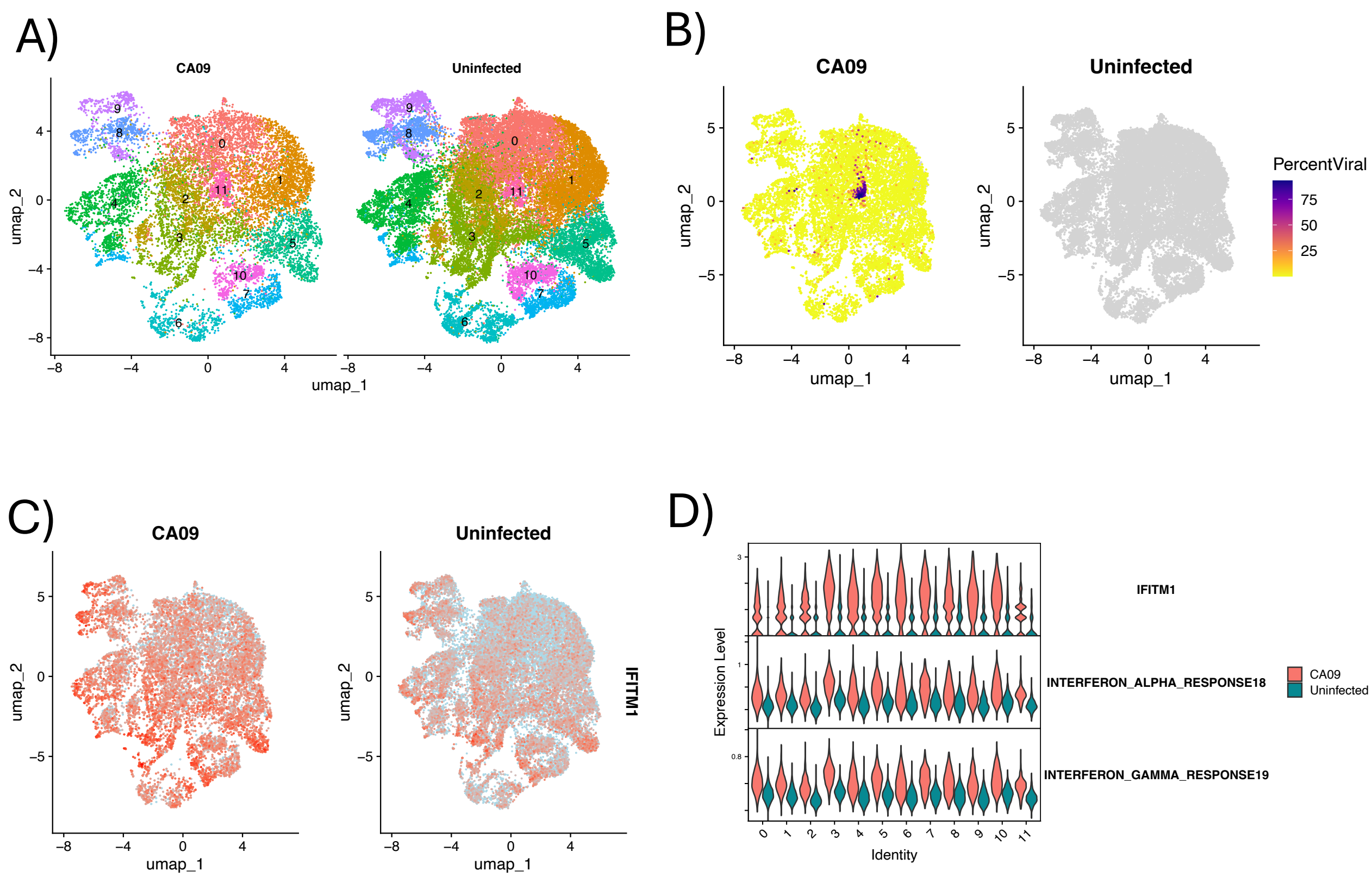

Fig. S4. IAV-induced divergent expression of two IFITM1 transcripts. **A**) Boxplot of raw read count difference between IFITM1-202 and IFITM1-201 in uninfected and IAV-infected conditions (p = 3e-13). Gray lines denoting matched LCLs. **B**) Boxplots of raw read counts for IFITM1-201 (left, p = 0.82) and IFITM1-202 (right, p = 1.8e–12) in Uninfected and IAV condition. Grey lines denoting matched LCLs.

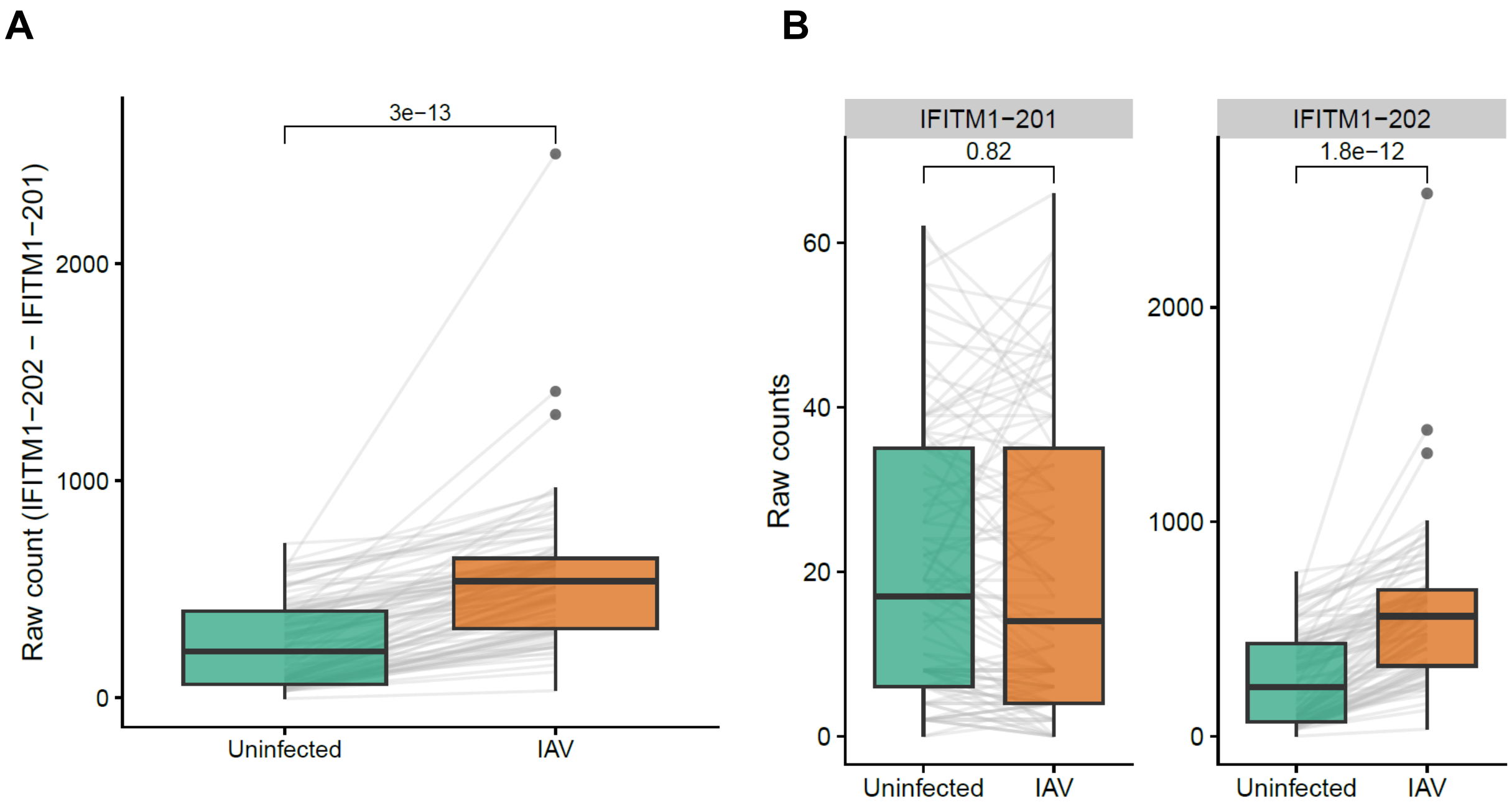

Fig. S5. A summary of sJunctions and their effects on splicing. An sJunction is defined as a splicing junction from Leafcutter that is associated to at least 1 sQTL. **A)** The sJunction counts for uninfected and IAV-infected LCLs from scHi-HOST. **B)** The number of sJunctions that have each category of effect for uninfected and IAV-infected LCLs.

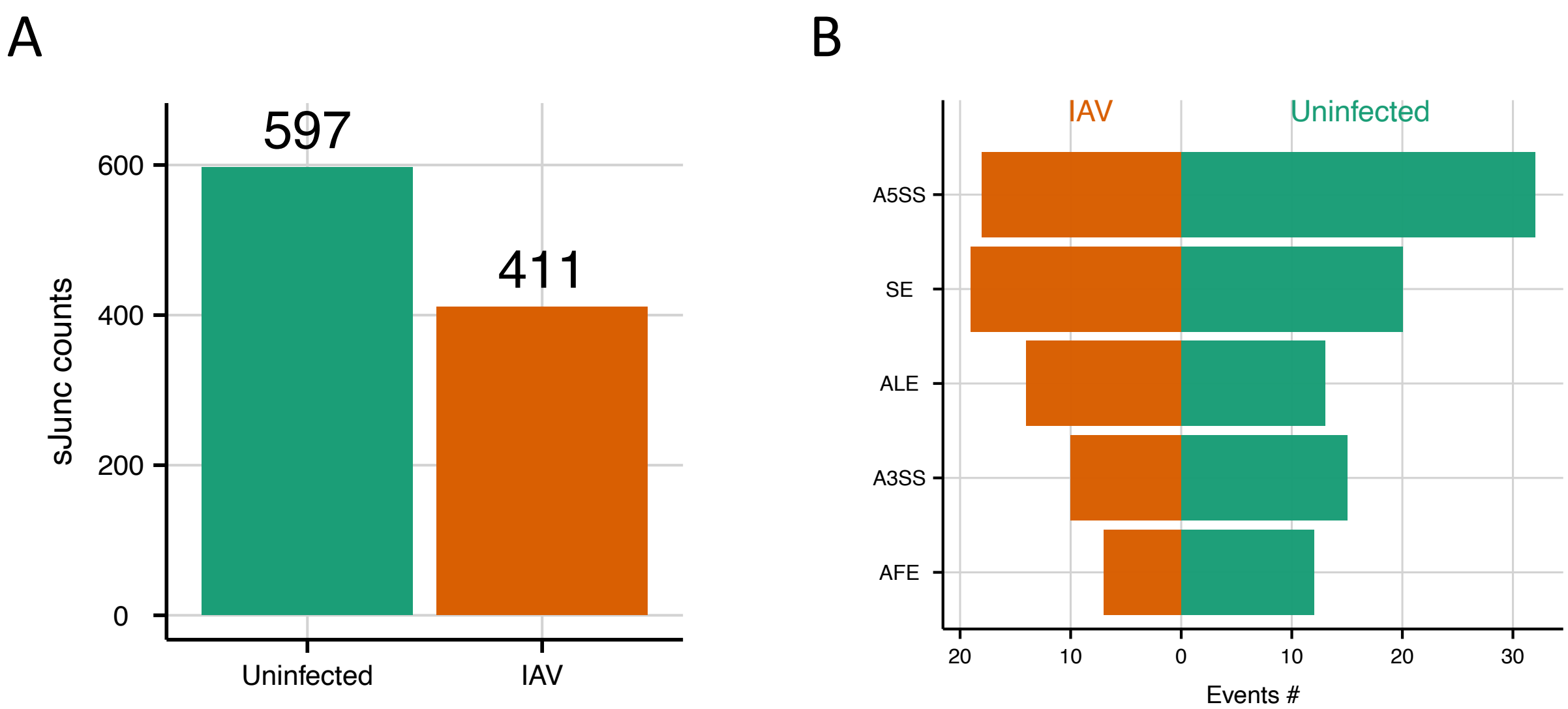

Fig. S6. Evaluation of power in the scHi-HOST sQTL pipeline. **A)** Mean expression of genes across LCLs vs. the probability of detecting a sQTL in both uninfected (top left) and IAV-infected conditions (top right). The probability of detecting sQTL was defined as fraction of genes in each bin associated with at least 1 sQTL (FDR < 0.05), 31,228 genes were tested for uninfected and 30,769 for IAV. **B)** The aggregated counts of sQTLs against average gene expression per bin for both uninfected (bottom left) and IAV-infected conditions (bottom right). Gene expression was transformed using log10(x+1).

A)

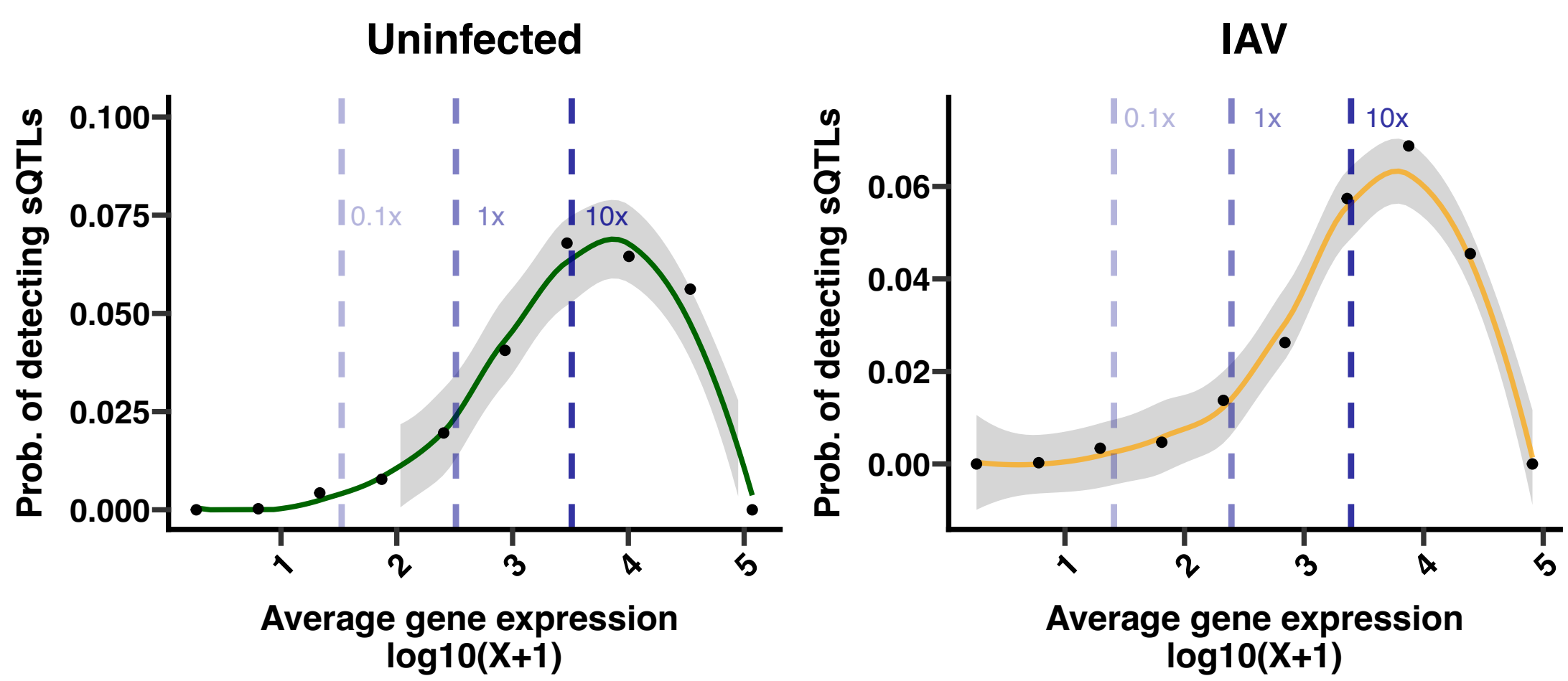

B)

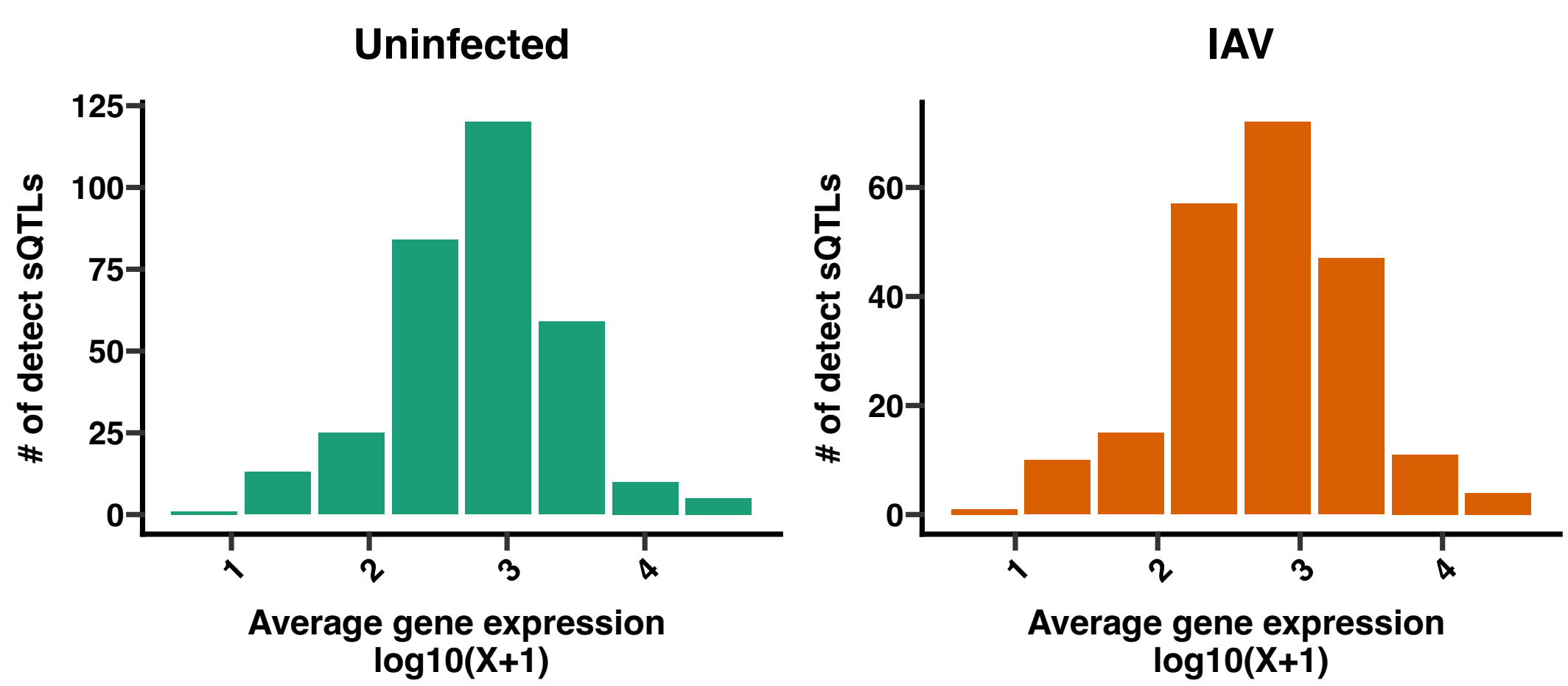

Fig. S7. Characterization of sQTLs based on their locations in a gene.

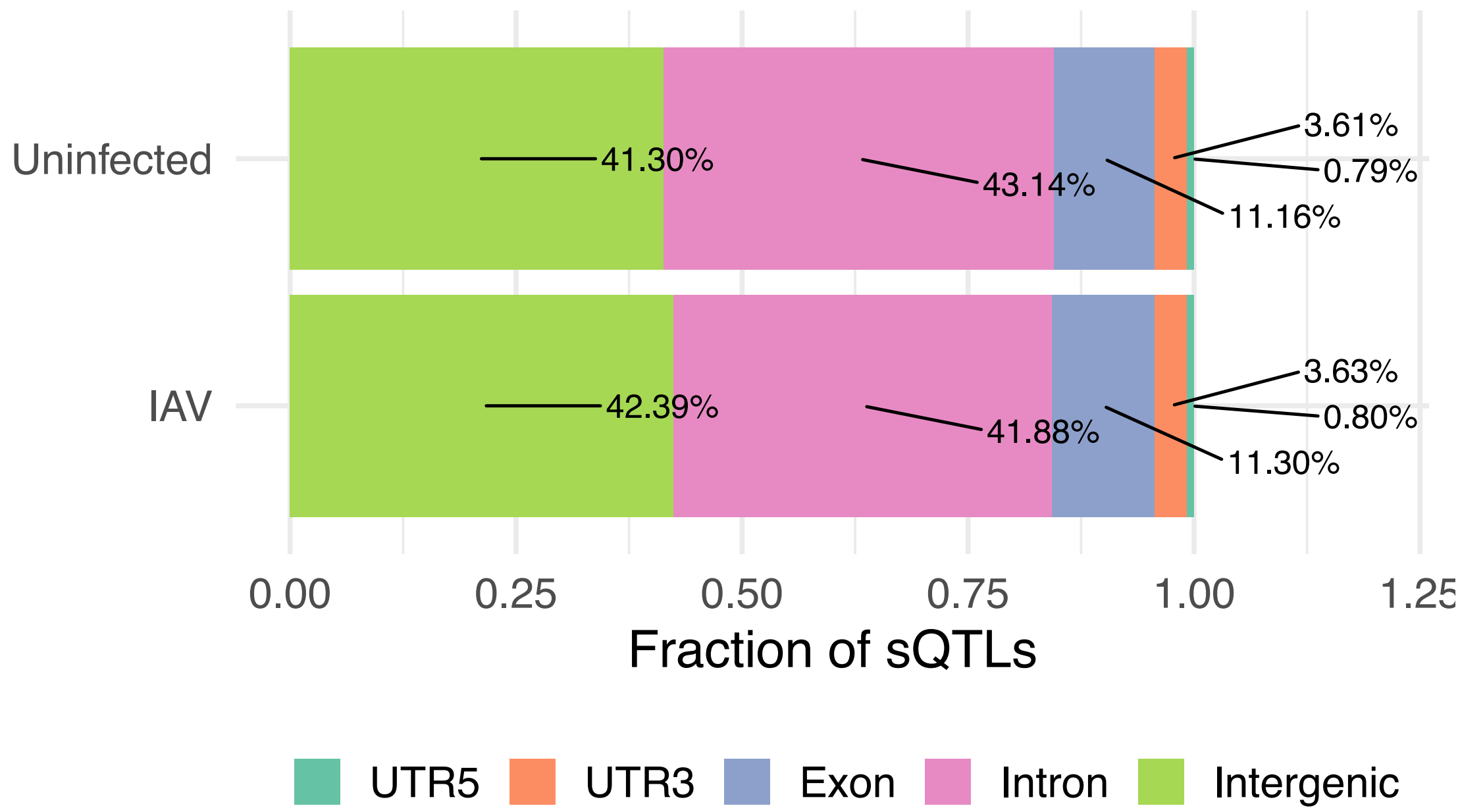

Fig. S8. Replication of scHi-HOST sQTLs in GTEx. The top panels show slope and p value comparison of significant sQTL-SNPs from the uninfected and IAV (y-axis) against GTEx EBV-infected LCL (x-axis). The bottom panels show slope and p value comparison of lead sQTL-SNPs per sJunction from the uninfected and IAV (y-axis) against GTEx EBV-infected LCL (x-axis).

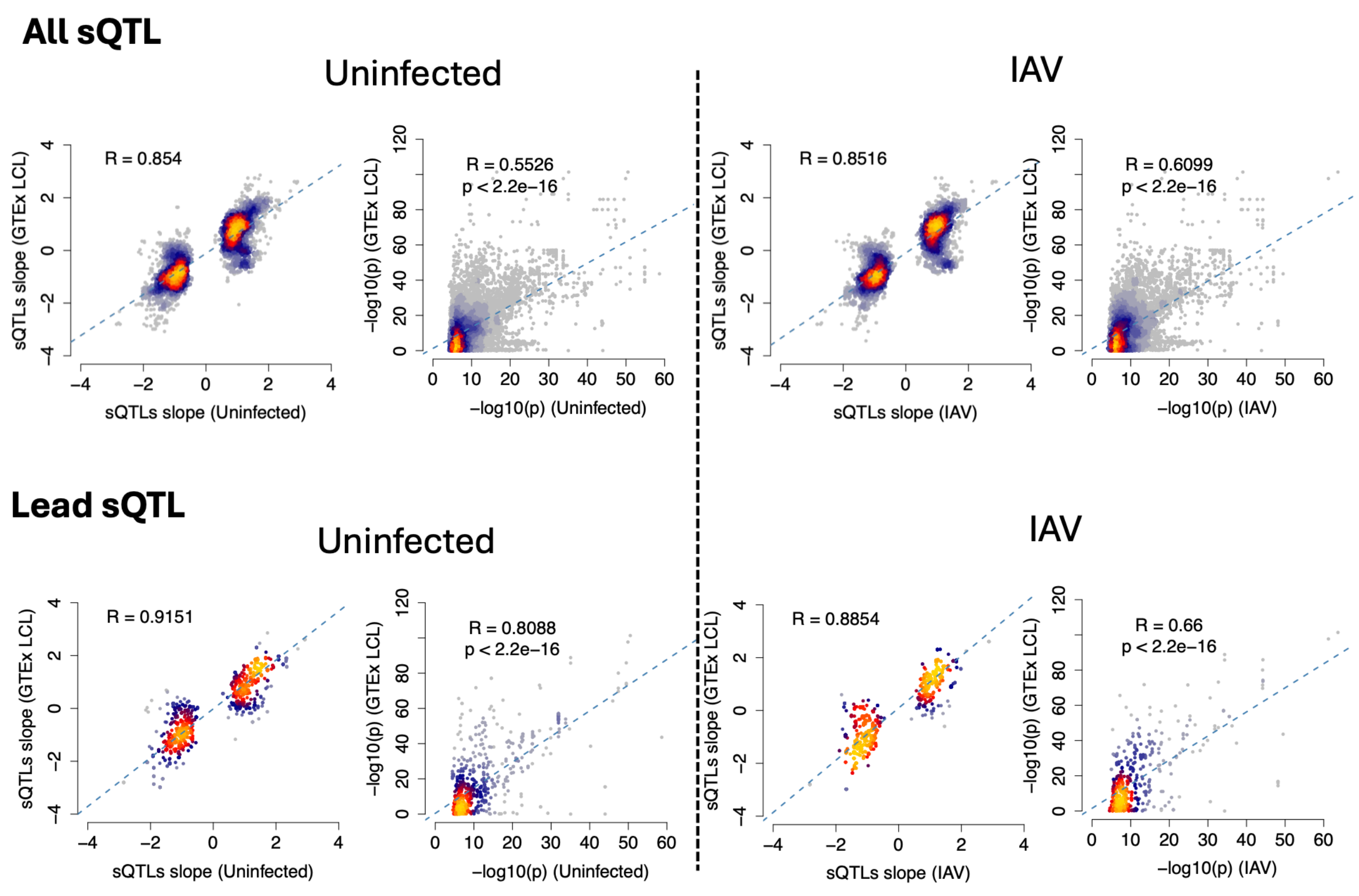

Fig. S9. Comparison of rsQTL identification by LRT vs. z-test. **A)** Scatter plot shows a significant positive correlation between interaction beta values from LRT and z scores from z-test. **B)** Same plots as in A), with points colored by rsQTL and csQTL defined by significant LRT (left; q-value < 0.1) and by significant Z-test (right; BH-corrected p-value < 0.05).

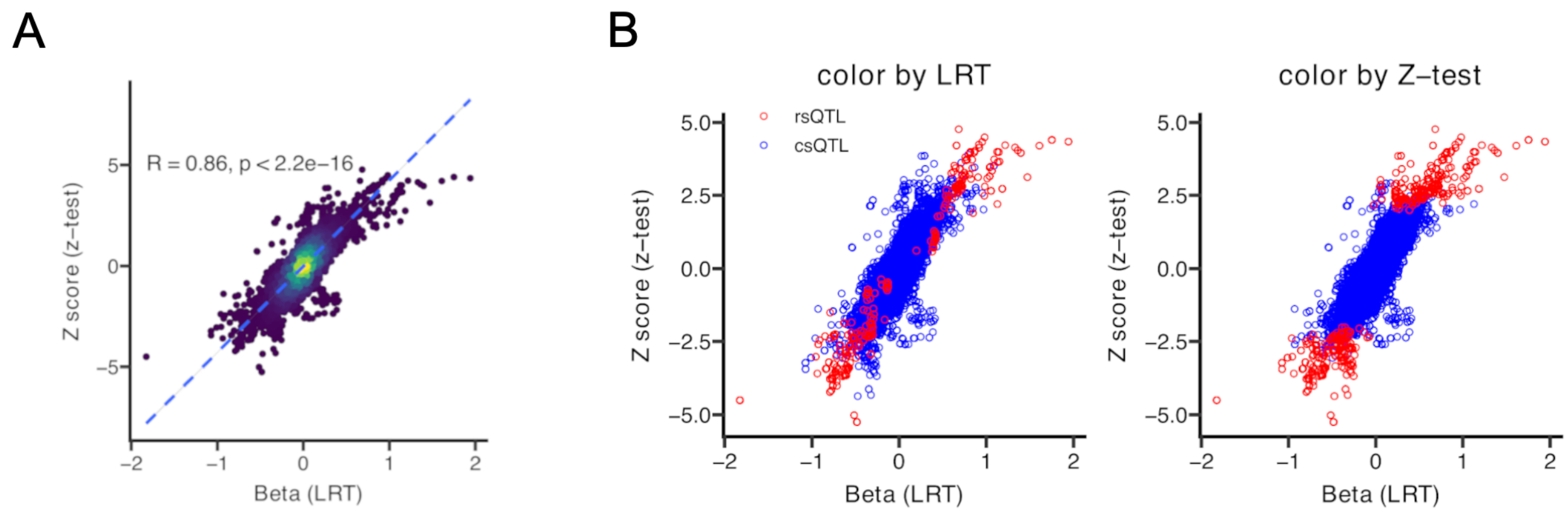

Fig. S10. LocusZoom plots of comparing *IFNAR2* in IAV sQTL against GWAS in severe/critical COVID19 (A2) (bottom) in **A**) and COVID19 B1 (hospitalized covid vs. not hospitalized covid) in **B**).

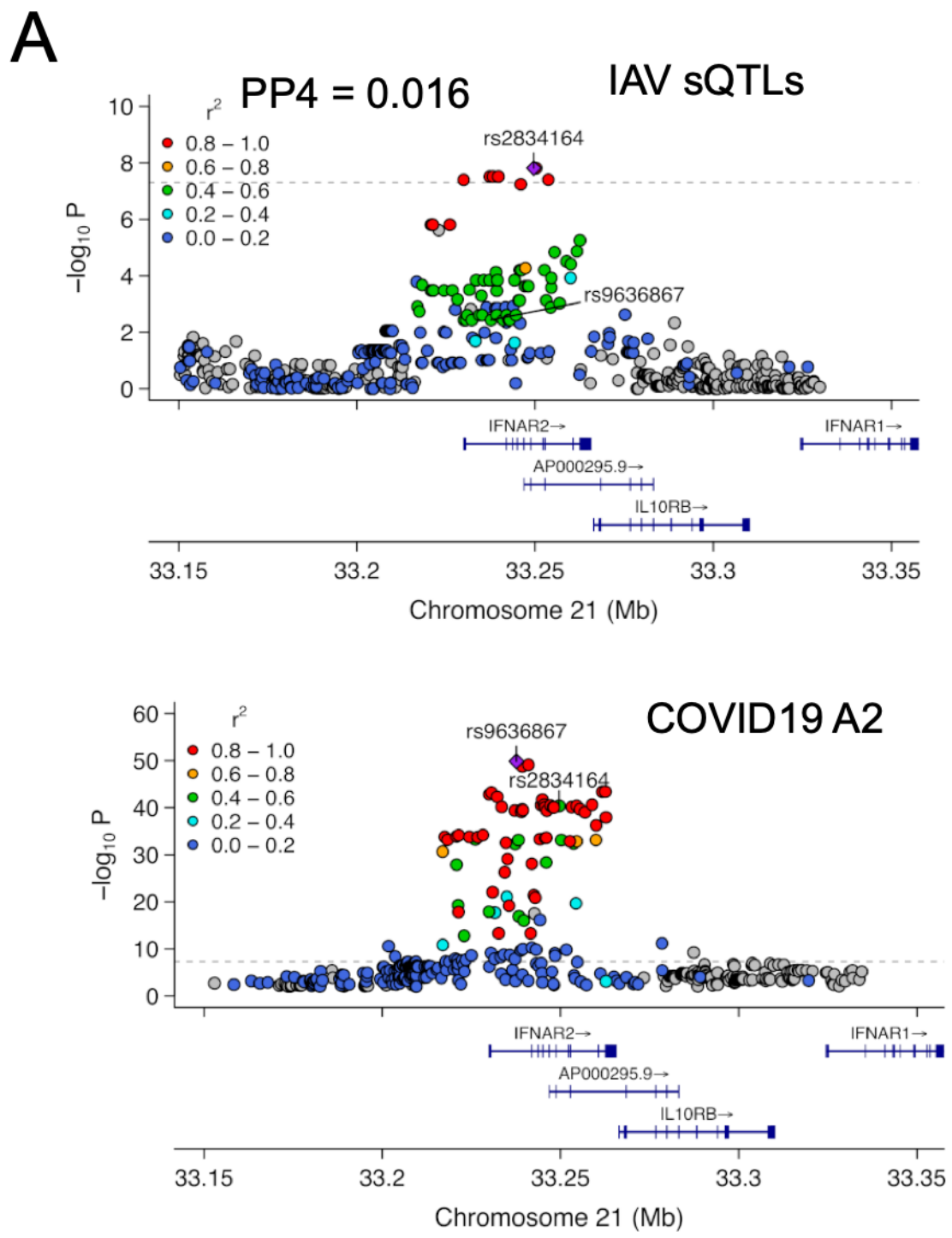

Fig. S11. Sashimi plot of U2AF1L4 rs17638853 with splice prediction from AlphaGenome.

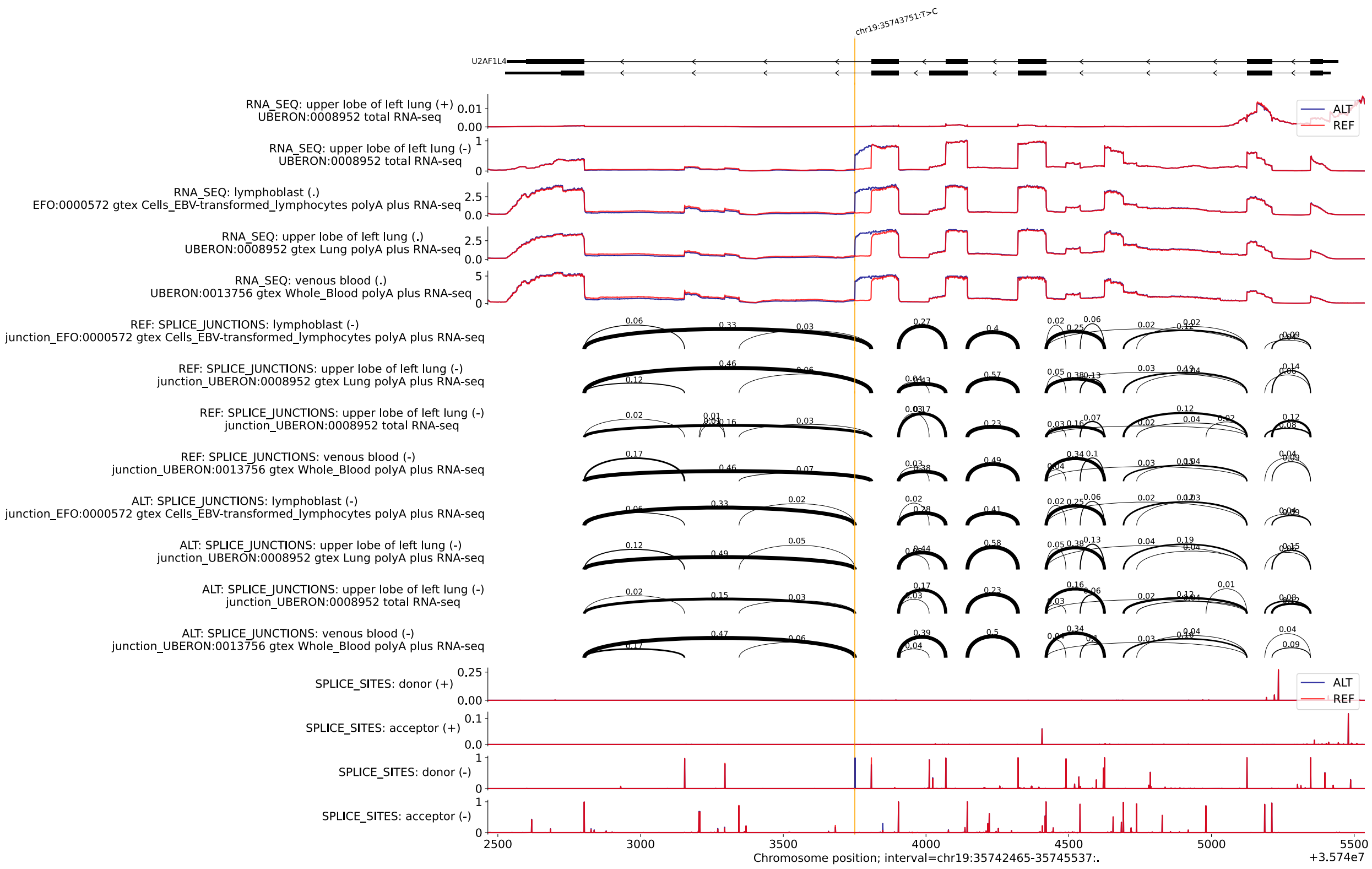

Fig. S12. Relative distance of sQTL for **A**) AI-predicted variants (either SpliceAI or Pangolin >= 0.2), **B**) lead variant per sJunction.

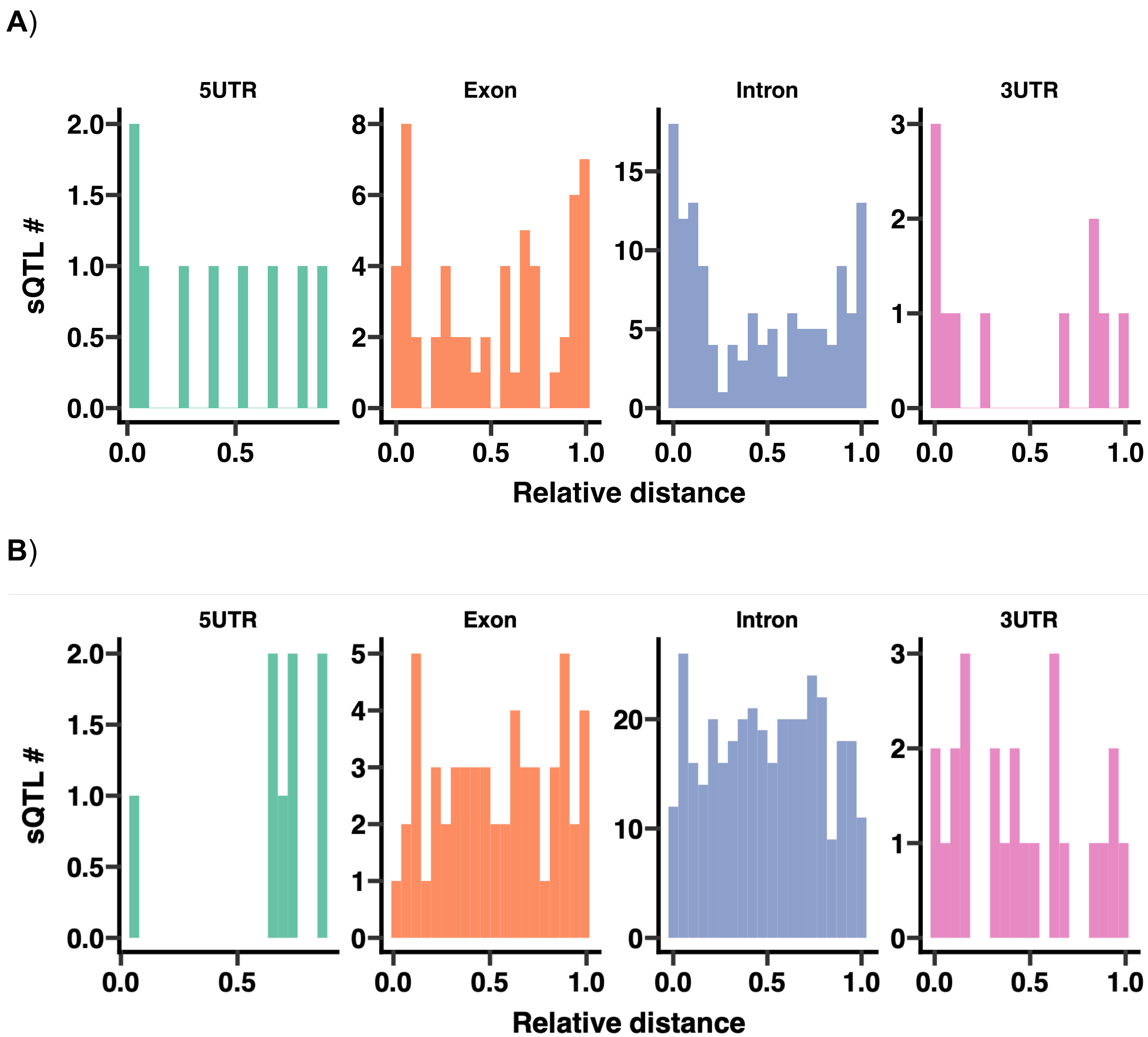

Figure S13: Comparison of SuSiE fine mapping with Pangolin score as prior against with default parameters. The concordant variants as shown in Figure 2E-G, 3E and 4H were colored in purple. The non-concordant variants (blue) between AI and SuSiE default from Fig. S14 were highlighted in blue.

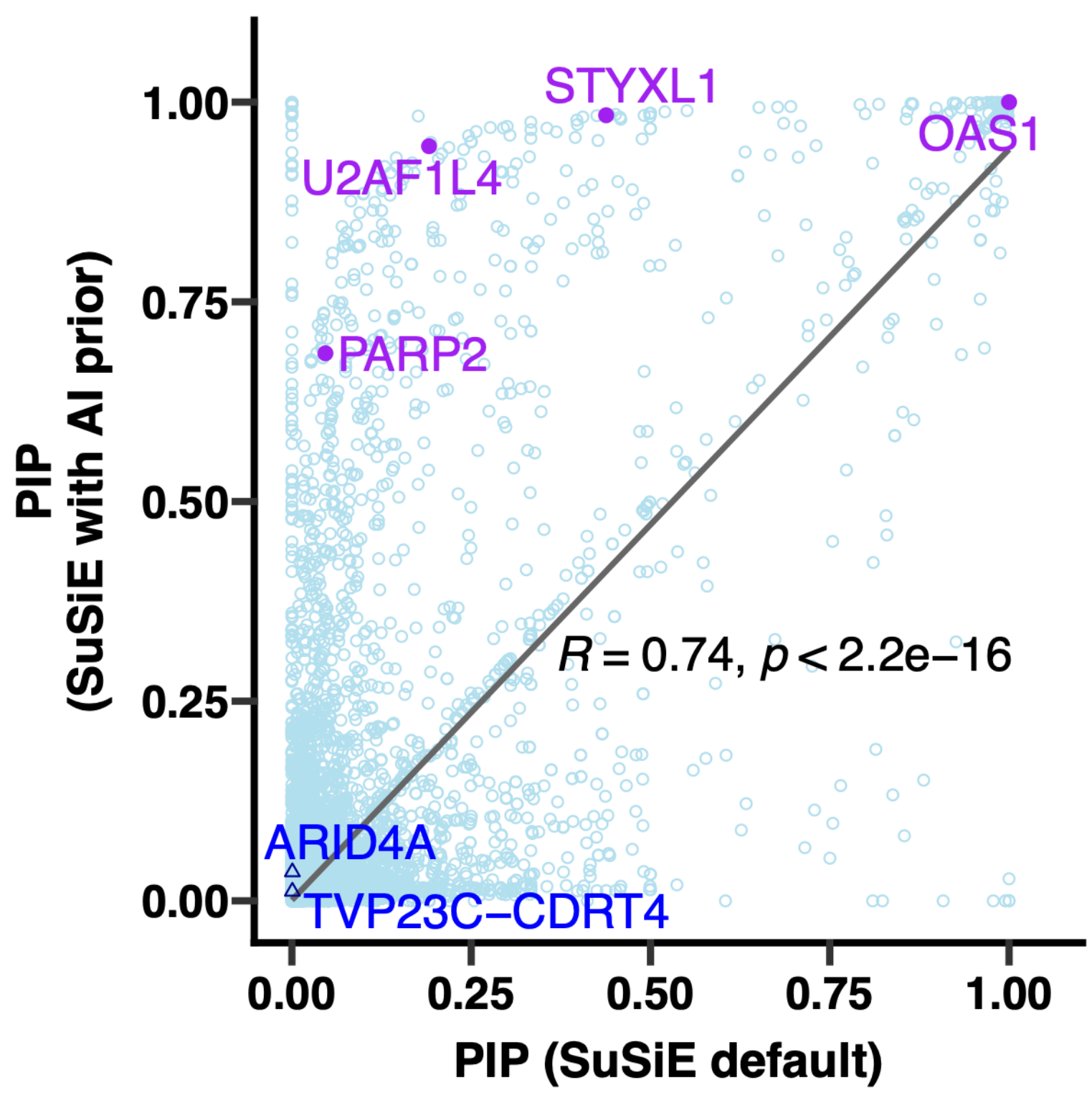

Fig. S14: Function enrichment of scHiHOST sQTL across different functional groups. Enrichments are shown for **A)** all combined uninfected and IAV sQTL, **B)** IAV response sQTL, **C)** IAV induced sQTL, and **D)** causal variants supported by both AI and SuSiE. The y-axis is odds ratio (observed/expected). Gray bars indicate not significant enrichments (adjusted p-value >0.05), whereas colored bars indicate significant enrichment after multiple-testing correction (adjusted p-value <0.05).

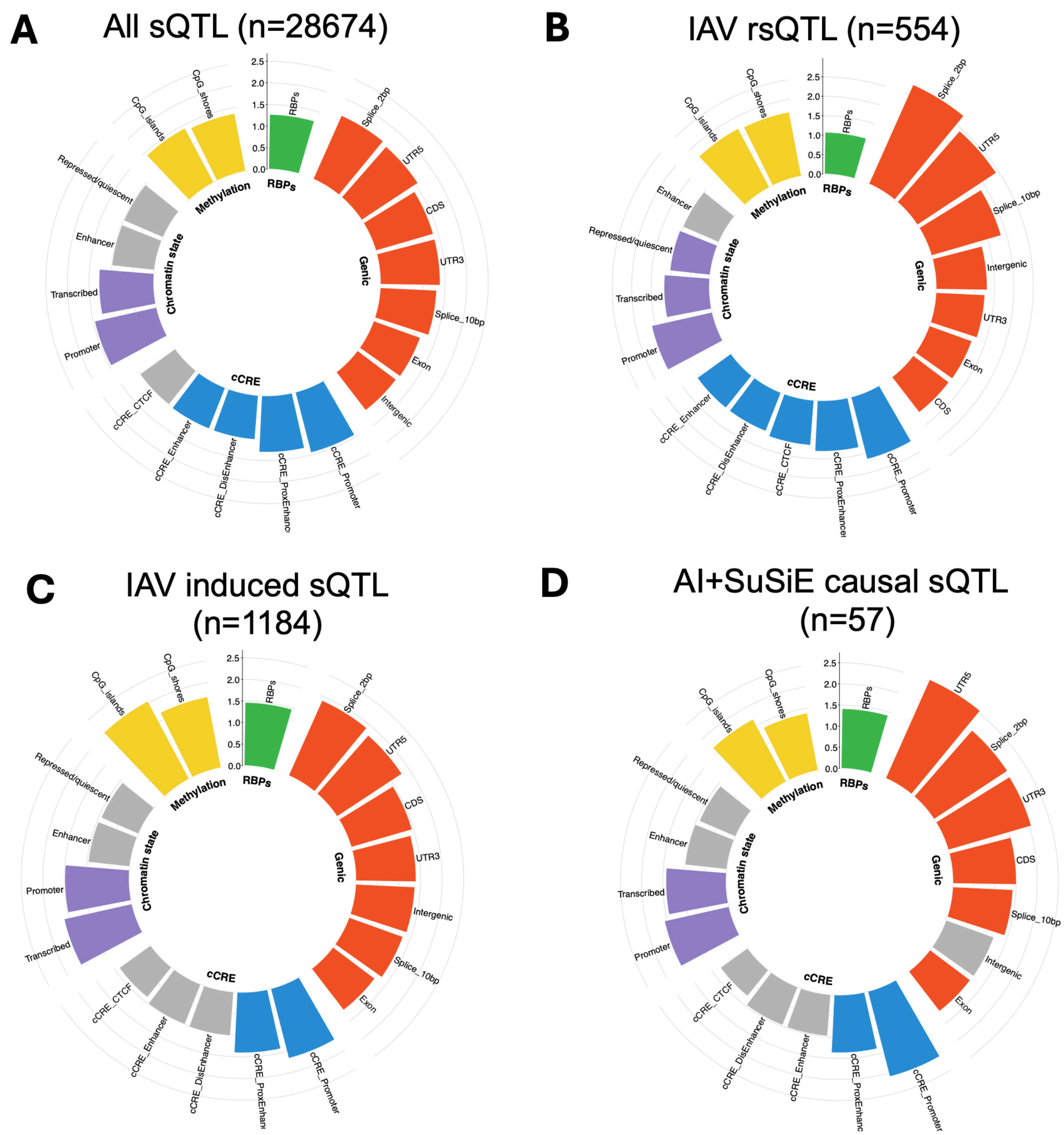

Fig. S15. LocusZoom plots of AI-predicted causal variants that do not fall within a SuSiE credible set, with top panel showing sQTL association, middle showing gene tracks with variants (purple arrow) and sJunction (red), and bottom panel showing SuSiE PIP scores. **A**) Example of rs1051861 from *ARID4A*, with its target splice junction in *PSMA3-AS1*. **B**) Examples of rs2302252 from *TVP23C-CDRT4*, with its target splice junction +133 bp in the same gene.

A) **B**)

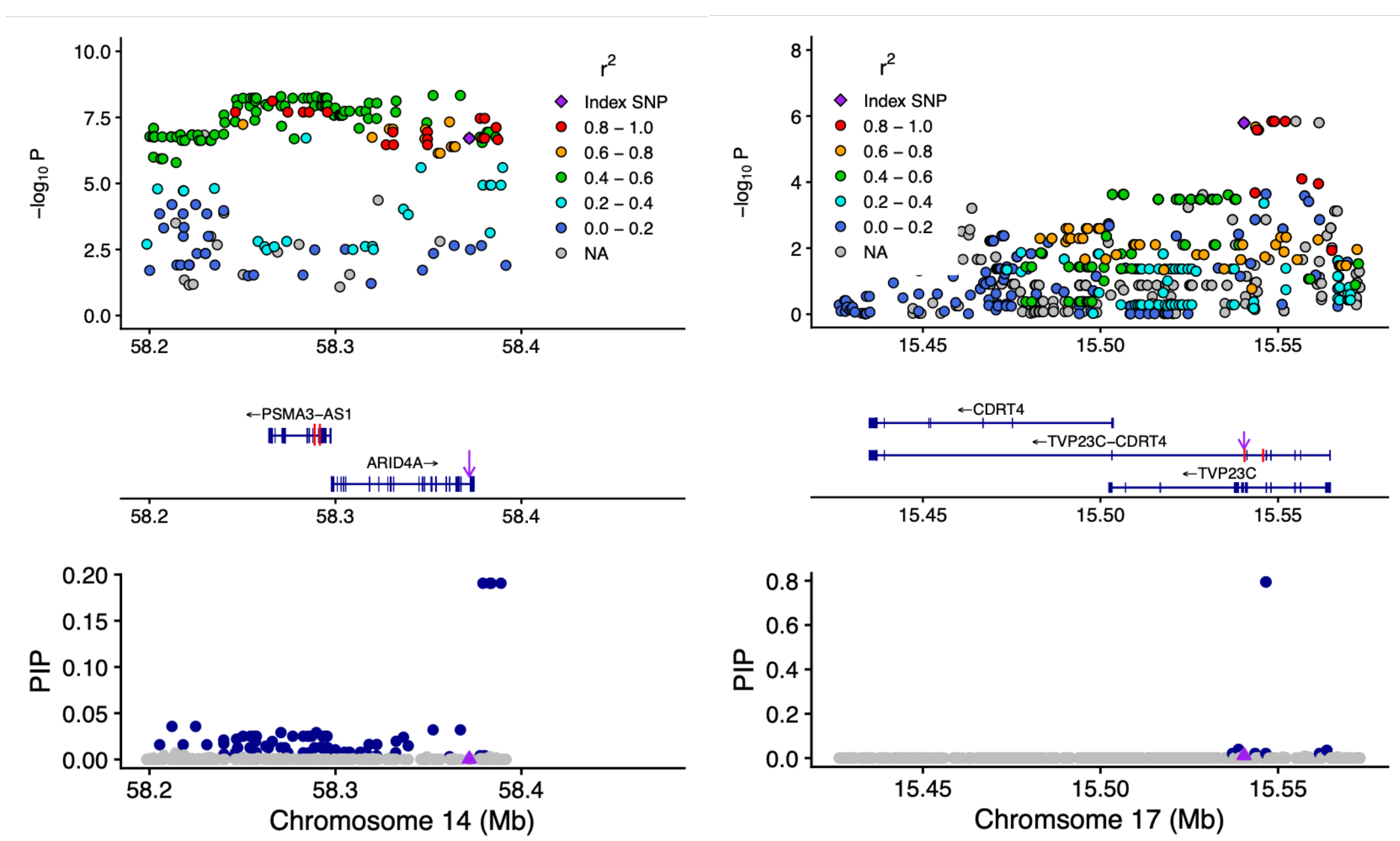

Fig. S16. Sashimiplot of PARP2 rs2297616 splice prediction from AlphaGenome.

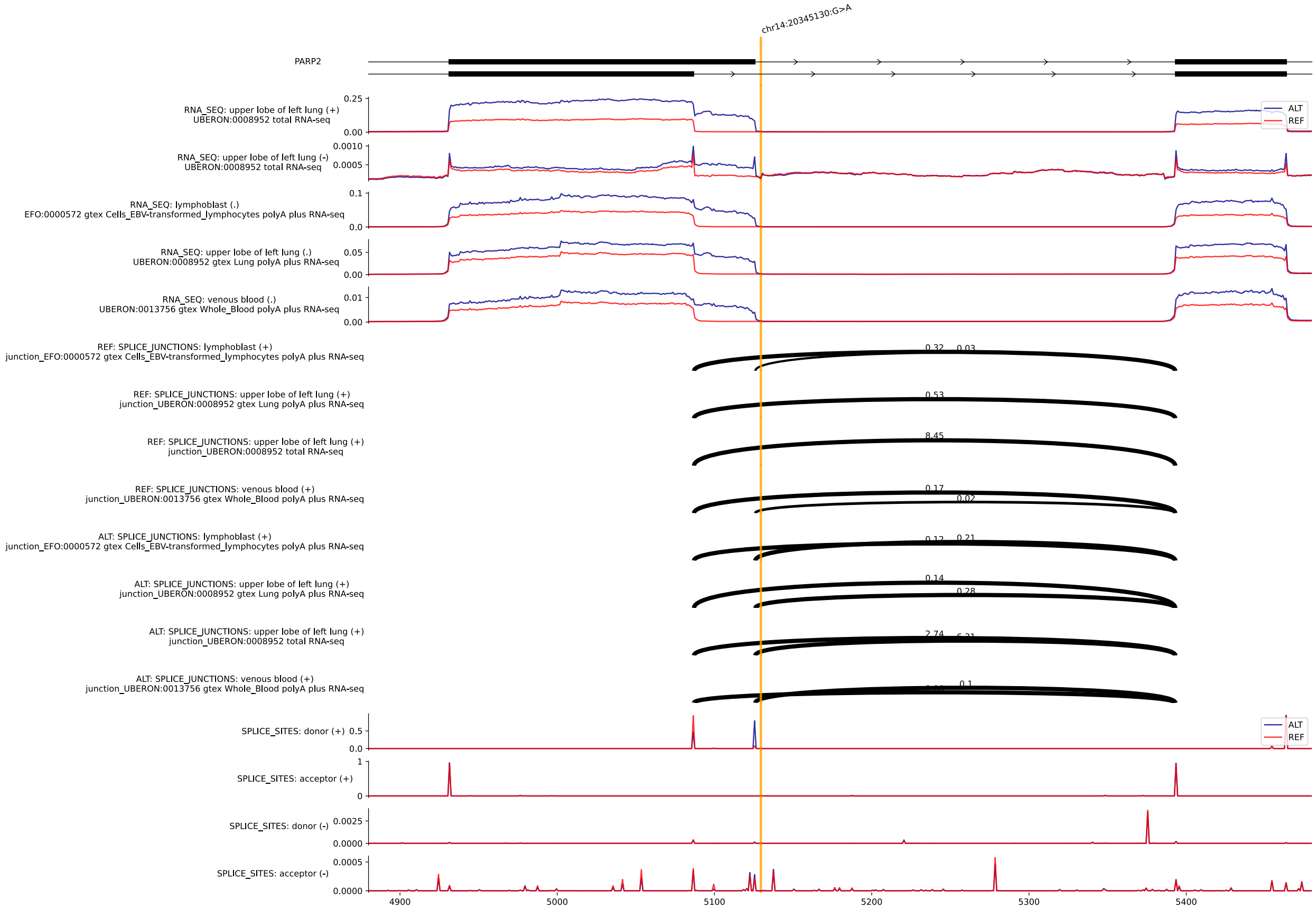

Fig. S17. Sanger sequencing validation of the PARP2 rs2297616 minigene splice reporter assay.

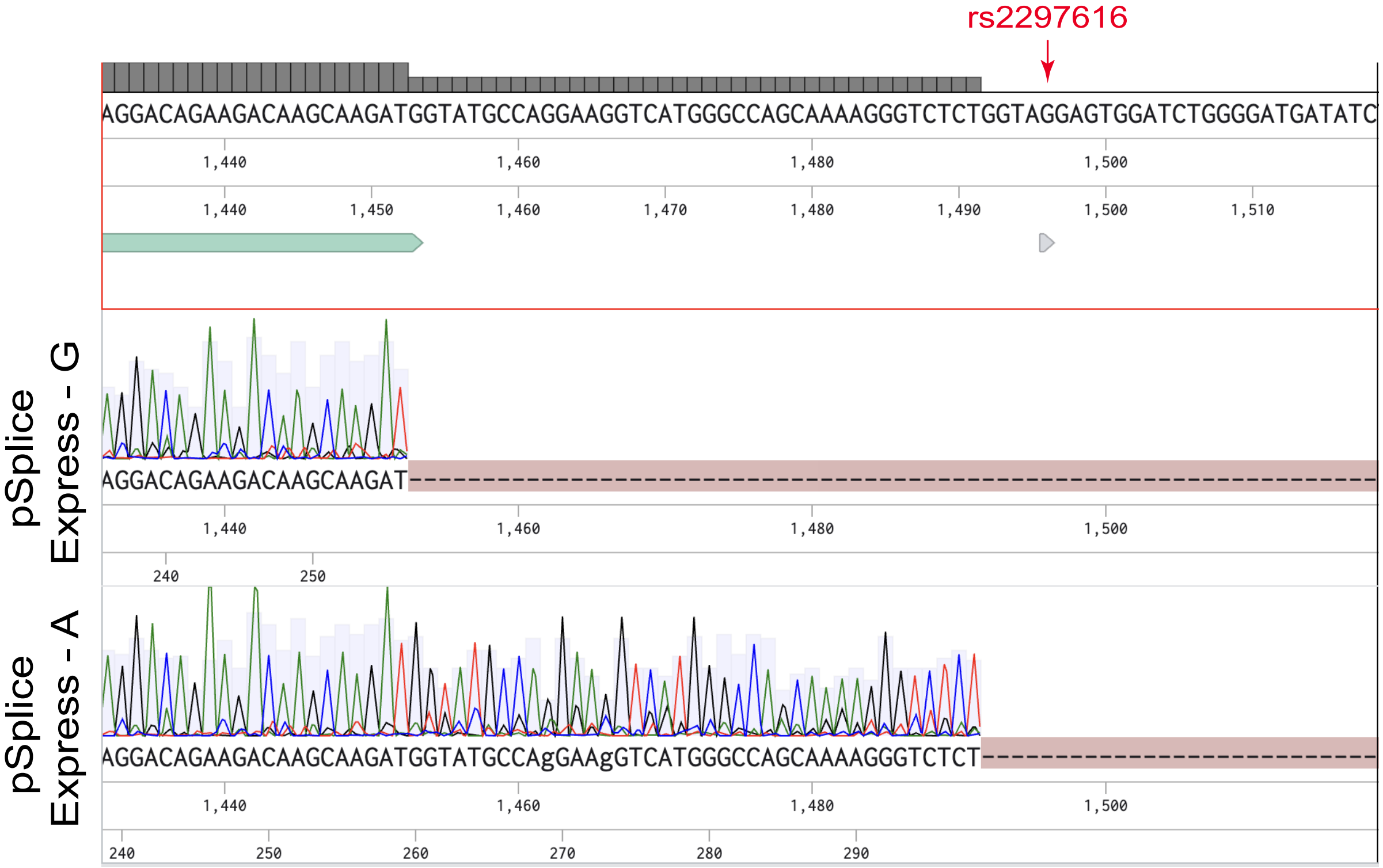

Fig. S18. Benchmarking sQTL detection across different co-variates. Correlation of PEER factors, sex, and genotypic PCs in Uninfected **A**) and in IAV-infected states **B**). We also benchmarked sQTL detection using sex, top two genotypic PCs as covariates, together with varying numbers of PEER factors (n=2, 3, 5, 10, 15). **C)** Number of significant sGenes identified at each PEER factor. **D)** Number of significant sQTL–junction pairs identified at each PEER factor. **E)** Ratio of sGenes shared with the PEER_3 (used in the main results) for each PEER factor. **F)** Ratio of sQTL–junction pairs shared with the PEER (n=3) analysis for each PEER factor. The number **G)** and ratio **H)** of detected causal sQTLs.

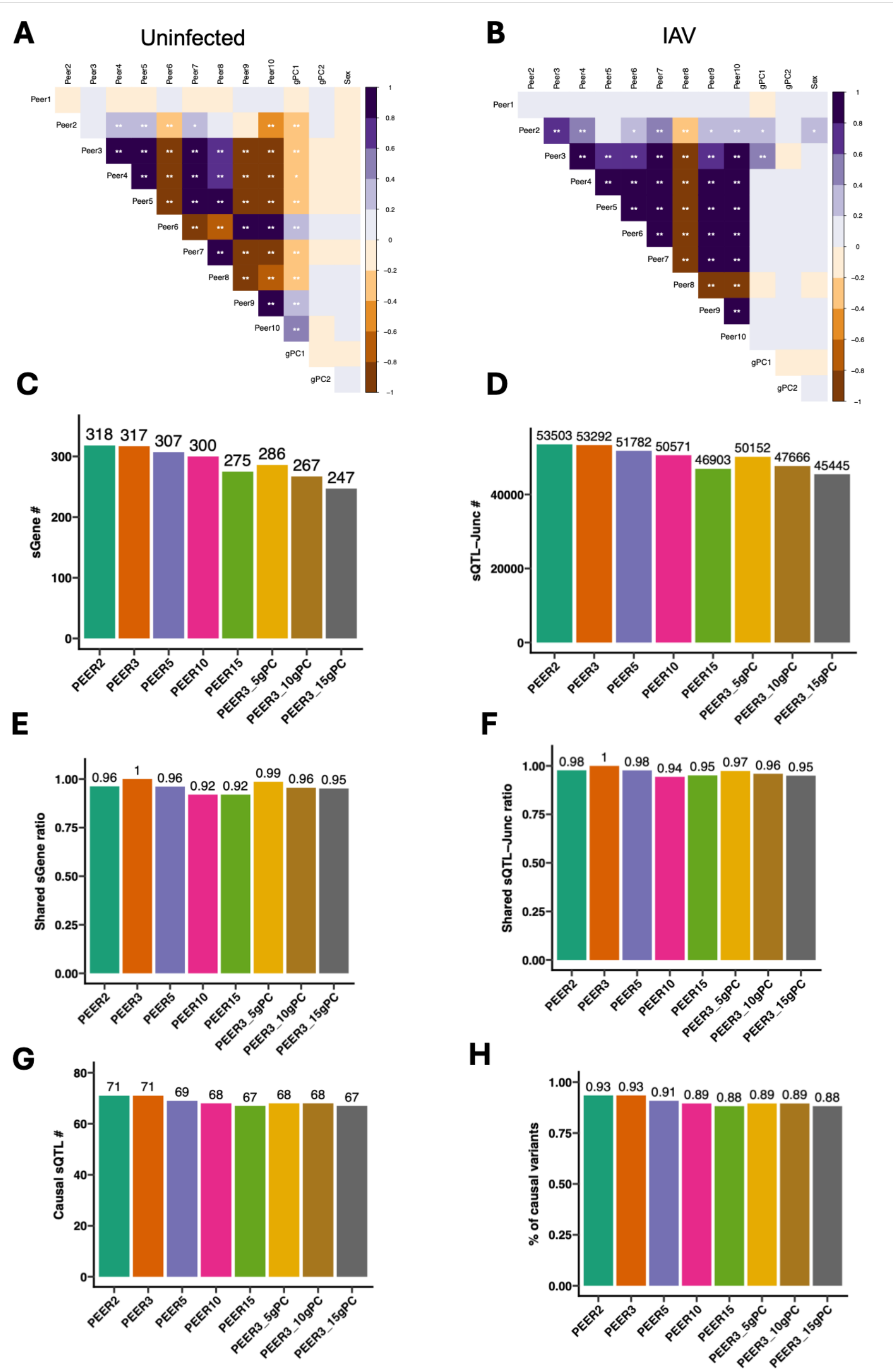

Fig. S19. Uncropped gel images. A) Uncropped image of Fig. 5D. **B)** Uncropped image of Fig. 5E. **C)** Uncropped image of Fig. 5F. **D)** Uncropped image of Fig. 5H.

A

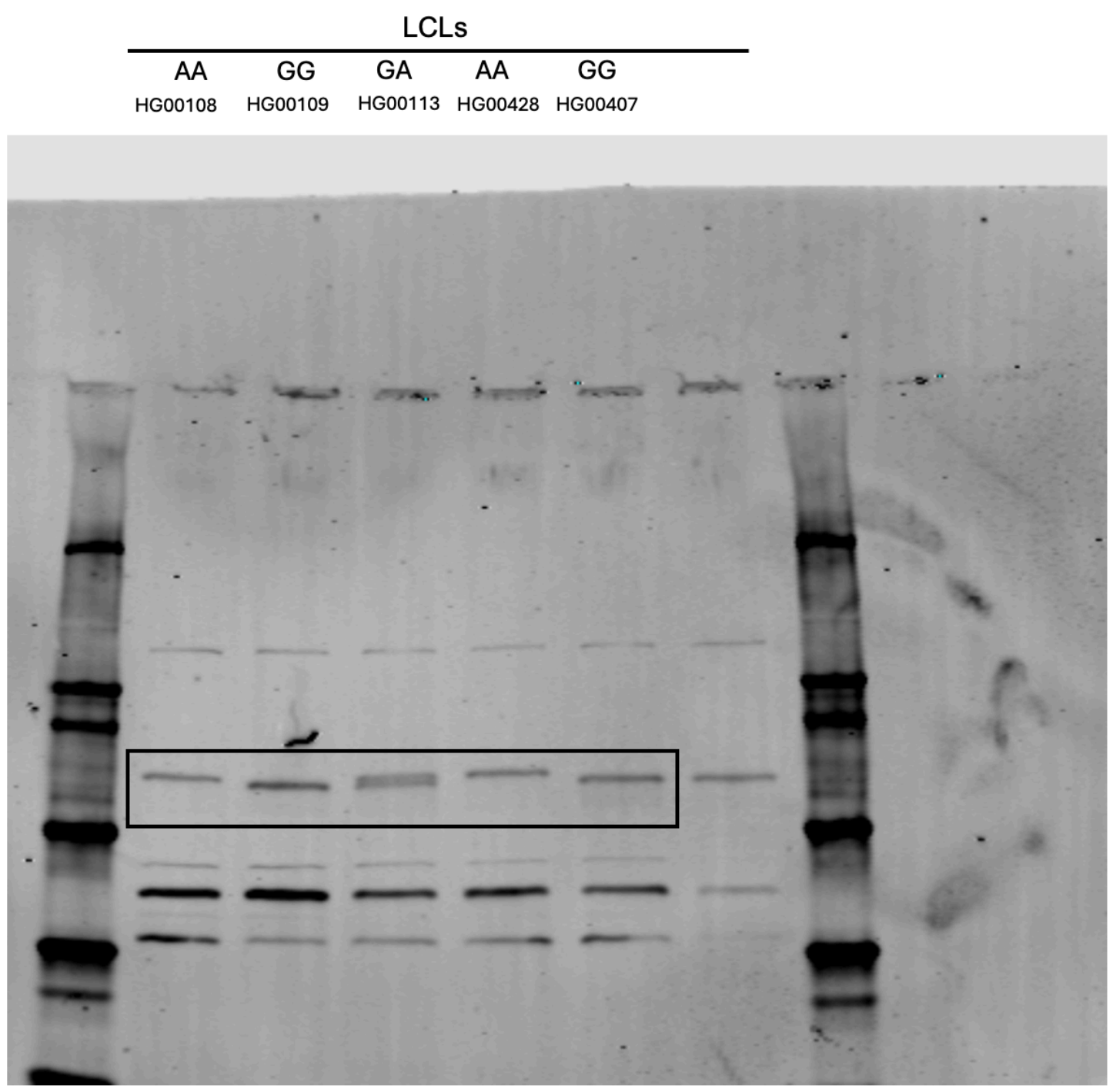

B

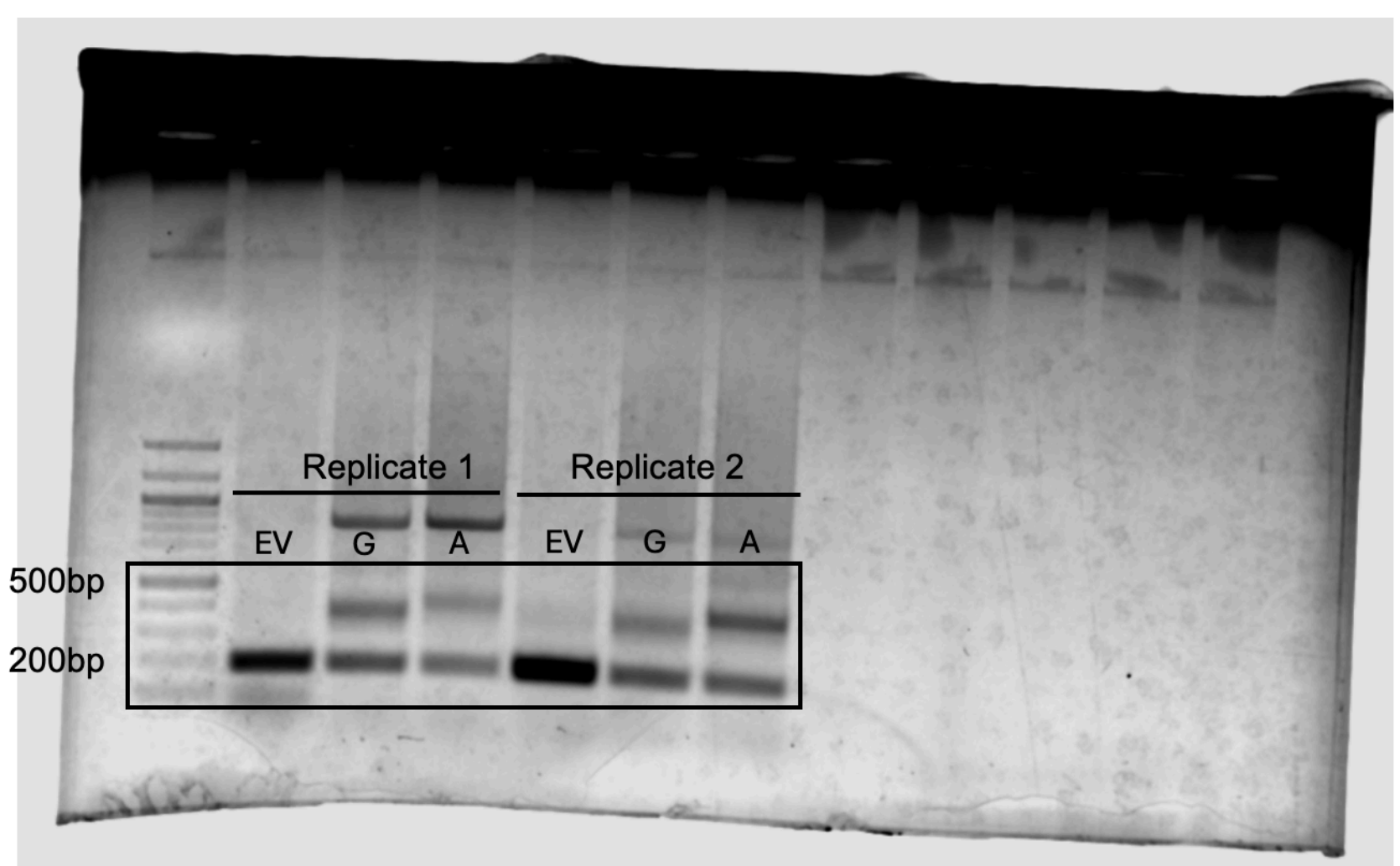

C

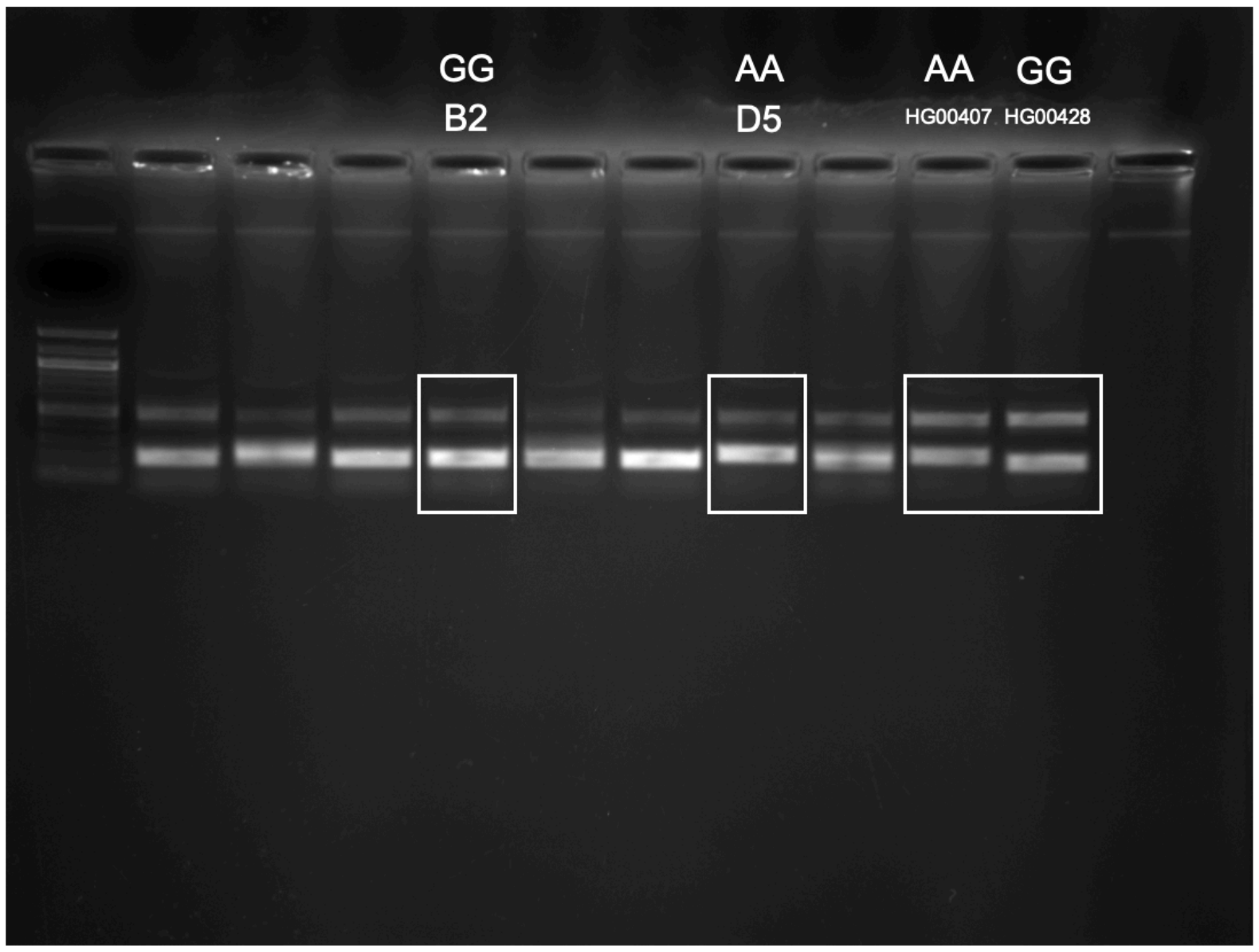
GG
B2
AA
D5
AA
HG00407
GG
HG00428

D

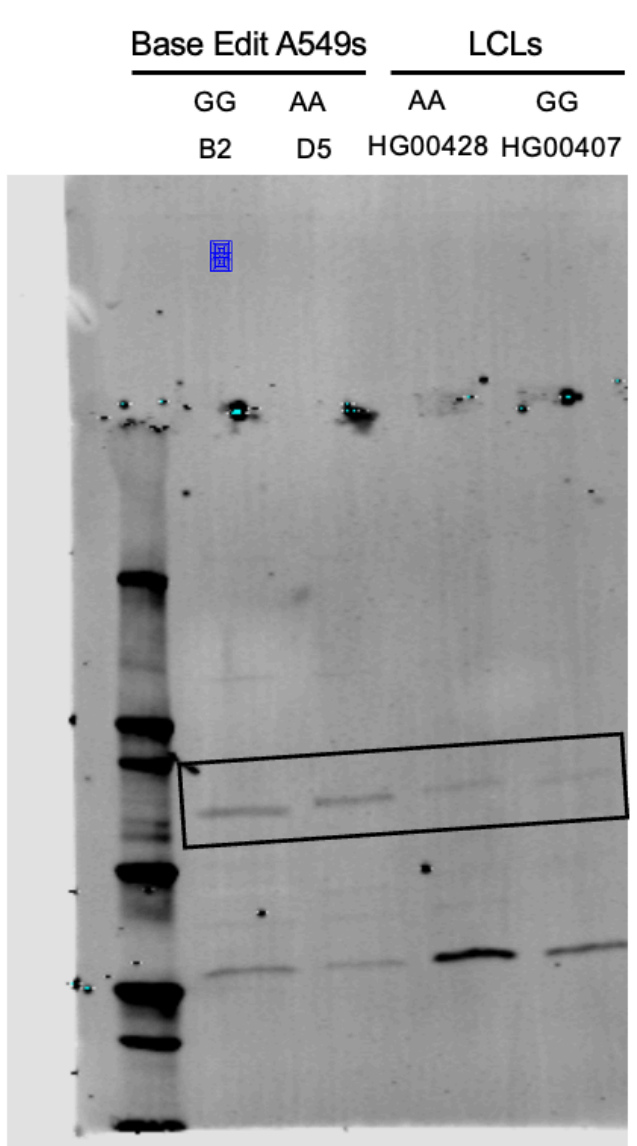
Base Edit A549s
LCLs
GG
B2
AA
D5
AA
HG00428
GG
HG00407

Table S10. Primers used for CRISPR-Cas9 editing of PARP2.

	**SEQUENCE 5' --> 3'**
**GRNA1**	AAAAGGGUCUCUGGUAGGAG
**GRNA2**	CUCUGGUAGGAGUGGAUCUG
**GRNA3**	UCUCUGGUAGGAGUGGAUCU
**HDR_G**	AGGAAGGTCATGGGCCAGCAAAAGGGTCTCTGGTAGGAGTCGATCT GCCGATGATATCTTGTTATTTCAACTCCTATTT
**HDR_A**	AGGAAGGTCATGGGCCAGCAAAAGGGTCTCTGGTAAGAGTCGATCT GCCGATGATATCTTGTTATTTCAACTCCTATTT
**ATTB1F**	GGGGCAAGTTTGTACAAAAAAGCAGGCTAATGGCAACACGGCTCCAGAAGAC
**ATTB2R**	GGGGACCACTTTGTACAAGAAAGCTGGGTCTTCCCCACCTTGGCTGTACACTCT
**RS2297616_F**	AATGGCAACACGGCTCCAGAAGAC
**RS2297616_R**	CTTGTTGTTGTTGAACTGGAGATTGG
